# Self-healing perovskite solar cells based on copolymer-templated TiO_2_ electron transport layer

**DOI:** 10.1038/s41598-023-33473-9

**Published:** 2023-04-19

**Authors:** Nakisa Lalpour, Valiollah Mirkhani, Reza Keshavarzi, Majid Moghadam, Shahram Tangestaninejad, Iraj Mohammadpoor-Baltork, Peng Gao

**Affiliations:** 1 Department of Chemistry, Catalysis Division, University of Isfahan, Isfahan, 81746-73441 Iran; 2 CAS Key Laboratory of Design and Assembly of Functional Nanostructures, and Fujian Provincial Key Laboratory of Nanomaterials, Fujian Institute of Research on the Structure of Matter, Chinese Academy of Sciences, Fuzhou, 350002 Fujian People’s Republic of China

**Keywords:** Energy science and technology, Materials science

## Abstract

Inorganic hole-transport materials (HTMs) such as copper indium disulfide (CIS) have been applied in perovskite solar cells (PSCs) to improve the poor stability of the conventional Spiro-based PSCs. However, CIS-PSCs' main drawback is their lower efficiency than Spiro-PSCs. In this work, copolymer-templated TiO_2_ (CT-TiO_2_) structures have been used as an electron transfer layer (ETL) to improve the photocurrent density and efficiency of CIS-PSCs. Compared to the conventional random porous TiO_2_ ETLs, copolymer-templated TiO_2_ ETLs with a lower refractive index improve the transmittance of input light into the cell and therefore enhance the photovoltaic performance. Interestingly, a large number of surface hydroxyl groups on the CT-TiO_2_ induce a self-healing effect in perovskite. Thus, they provide superior stability in CIS-PSC. The fabricated CIS-PSC presents a conversion efficiency of 11.08% (Jsc = 23.35 mA/cm^2^, Voc = 0.995, and FF = 0.477) with a device area of 0.09 cm^2^ under 100 mW/cm^2^. Moreover, these unsealed CIS-PSCs retained 100% of their performance after aging tests for 90 days under ambient conditions and even increased from 11.08 to 11.27 over time due to self-healing properties.

## Introduction

Perovskite solar cells (PSCs) have attracted significant attention over the past few years concerning the application of clean and renewable energy^1–7^. The unique characteristics of optoelectronic perovskite solar cells (PSCs), such as high absorption coefficient, suitable absorption wavelengths in UV and visible light regions, and direct bandgap, have encouraged research on these cells^8–15^. Over time, the power conversion efficiency (PCE) of PSCs has increased from 3.8 to 25.7%^16,17^. A conventional perovskite solar cell possesses the configuration of Glass/FTO/compact TiO_2_/nanocrystalline random porous TiO_2_ (RP-TiO_2_)/MAPbI_3_/Spiro-OMeTAD/Au. In this type of solar cell, the optical photons are first absorbed by the perovskite absorbent layer, followed by the generation of coulombically bounded electron–hole pairs. The electrons move to the electron transport layer (ETL), and the holes enter the hole transport layer (HTL) from the opposite side. Finally, the electrons exit the system through metal electrodes, arrive at the external circuit, and establish an electric current. PSCs must have good stability along with good efficiency for commercial purposes^16^. In this regard, moisture^17,18^, UV light^19,20^, oxygen^21^, and temperature^17–21^ effectively make PSCs unstable and damage them^22^. CH_3_NH_3_PbI_3_ perovskite interaction with H_2_O molecules and degradation to PbI_2_, HI, and CH_3_NH_2_ is a reason for the instability of PSC. Photocatalytic degradation of perovskite materials, especially in the presence of an anatase TiO_2_, is the reason for the instability of PSC against UV light. Oxygen diffusion into the MAPbI_3_ lattice and O_2_^−^ production against light leads to the deprotonation and destruction of perovskite. MAPbI_3_ can be degraded at 85 °C while kept in an inert atmosphere as well.

Furthermore, although some additives such as lithium salts (Li-TFSI) and 4-tert-butyl pyridine (TBP) are added to Spiro-OMeTAD to increase the conductivity of this HTL, the lithium salt is hydrophilic, destroys the perovskite layer over time, and reduces the stability of the cells^23,24^. Some inorganic compounds such as CuSCN^25,26^, NiO^27^, CuGaO_2_^28^, CuInS_2_^29^, CuInSe_2_^30^, Cu_2_ZnSnS_4_^31,32^ and Cu(In,Ga)(S,Se)_2_^31,33^ can replace Spiro-OMeTAD since they are cheaper, easier to synthesize and more stable against moisture. Among the above semiconductors, CuInS_2_ has been used more in solar cells due to its excellent properties, such as low toxicity and suitable bandgap^29,33^.

Despite their attractive features, CuInS_2_-based PSCs (CIS-PSCs) currently present lower PCE in comparison to their common counterpart^29,34–36^. Lv et al. used CuInS_2_ quantum dots (QD) as HTL in PSC with the configuration of TiO_2_ (mesoporous)/CH_3_NH_3_PbI_3_/HTL/Au, and modified the surface of CuInS_2_ QDs by cation exchange to improve the carrier transport. CuInS_2_ QDs with a ZnS shell layer were capable of achieving an efficiency of 8.38%^31^. The highest efficiency reported so far for these cells is known to be 10.85%^29,37^. However, only very few reports exist on CIS-PSCs^31,35,39^. Hence, developing novel strategies, which provide the construction of highly efficient and stable CIS-PSCs, is necessary. One approach to increase the efficiency of this type of cell could be the substitution of general ETL RP-TiO_2_ with copolymer-templated TiO_2_ (CT-TiO_2_) compounds. The template-supported approach is one of the facile and low-cost routes for preparing mesoporous films. This method uses three-block copolymers such as P123 and F127 as templates. These block copolymers are removed from the inorganic framework hybrid film after the growth of the material to generate pores^38,39^. Compared to the RP-TiO_2_ layer, a CT-TiO_2_ film provides a larger surface area, allowing more perovskite uptake. Moreover, the porous structures obtained using these templates exhibit a highly progressive impact on the transparency of the coated film^40–42^. Thus, in parallel with the higher transparency, given the regular interconnection of all pores and higher perovskite loading, these compounds could result in higher photocurrent density and improved efficiency^43–45^. Furthermore, utilizing a simple method to prepare a solution and a dip-coating method to fabricate ETL is very promising regarding commercialization. However, rigorous research is required to explore further the advantages of template-assisted mesoscopic construction in PCE and the stability of CIS-PSCs to fulfill the demands for the commercialization of this strategy. For example, perovskite self-healing can be one of the properties of these template-assisted ETLs due to bearing high surface hydroxyl groups.

In the present study, P123 triblock copolymer has been used as the template for a titanium dioxide sol–gel solution to fabricate a novel smooth, low refractive index and transparent ETL for improving the efficiency and stability of the CuInS_2_-based PSC cells. In this regard, the dip coating method was applied to prepare the CT-TiO_2_ ETLs. According to morphological and structural studies, anatase TiO_2_ ETLs were highly transparent, uniform, and pinholes free. The fabricated CIS-PSC based on CT-TiO_2_ yielded a conversion efficiency and a high photocurrent efficiency of 11.27% and 23.35 mA/cm^2^, respectively. Remarkably, unsealed CIS-PSCs showed excellent performance in stability tests during 90 days under ambient conditions. Strikingly, the perovskite coated on the templated TiO_2_ showed self-healing property under vapor spray. Moreover, the perovskite film and the corresponding devices could rapidly recover to their original phase and performance after exposure to vapor spray and drying in ambient air. As a result of their self-healing properties, the unsealed CIS-PSCs-based CT-ETL retained 100% of their efficiency after 90 days of aging tests under ambient conditions and even improved from 11.08 to 11.27% over time. Based on the fabricated CT-TiO_2_ films, this ETL material suggests considerable potential to be used in stable mesoporous PSCs.

## Results and discussion

### Characterization of CT-TiO_2_ layers

CT-TiO_2_ films with different thicknesses were prepared using a P123 block copolymer via the sol–gel process. In this regard, the role of this novel ETL on the light transmittance and photovoltaic performance in CIS-PSCs was investigated.

**Figure 1:**
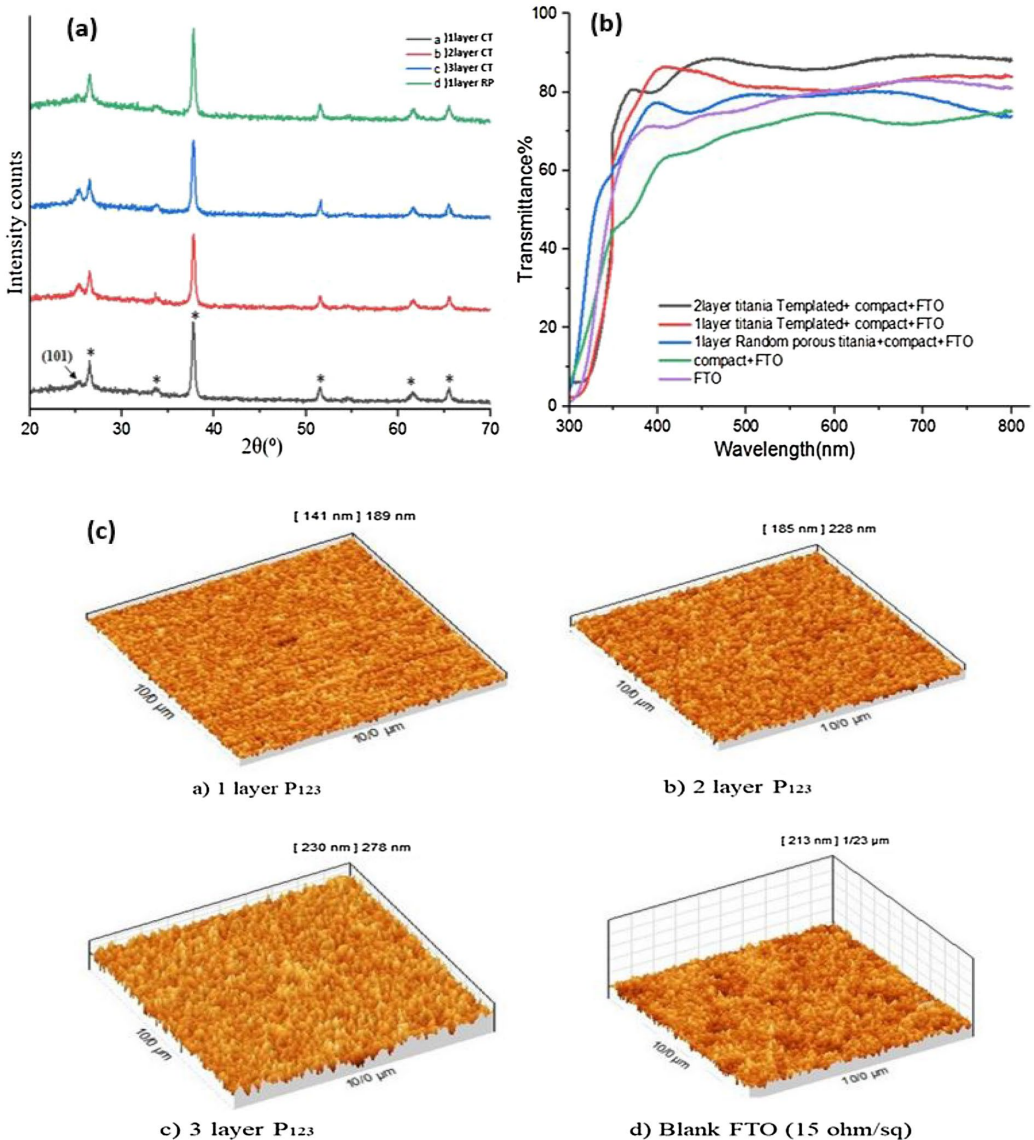
(**a**) XRD patterns of TiO_2_ layers, black) FTO/1 layer CT-TiO_2_, red) FTO/2 layer CT-TiO_2_, blue) FTO/3 layer CT-TiO_2_, and green) FTO/1 layer RP-TiO_2._ The symbol (*) corresponds to the peaks of the FTO substrate. (**b**) Transmittance spectra of TiO_2_ layers coated on FTO with different configurations. (**c**) AFM of CT-TiO_2_ layers placed on the FTO (15 Ω/sq).

Figure 1a illustrates the XRD patterns of the CT-TiO_2_ and RP-TiO_2_ films coated on the FTO substrate. The TiO_2_ anatase peak of the (101) plane is observed at 25.28°. As the number of layers increases, the peak intensity grows. The anatase crystal sizes were estimated to be around 28.37, 19.54, 15.97, and 49.92 nm, using the Debye–Scherrer equation, corresponding to CT-TiO_2_ of one layer, two layers, three layers, and RP-TiO_2_ film, respectively. In fact, a CT-TiO_2_ film is composed of a semi-crystalline network, including a significant amount of amorphous titania and anatase nanocrystals^46^. Therefore, the small amount of anatase crystalline phase could result in less UV light absorption by CT-TiO_2_ film. Therefore, a more stable cell could be obtained compared to RP-TiO_2_ cells.

Figure 1b shows the transmittance spectra of FTO-coated glasses in the presence of CT-TiO_2_ films with different thicknesses. These CT-TiO_2_ films increase the visible light transmission of the FTO glass substrate. Moreover, the compact TiO_2_ and RP-TiO_2_ *meso-*film, generally used for PSCs, show less transmittance than CT-TiO_2_ films. This can be attributed to the higher porosity of the former and, namely, a lower refractive index of the CT-TiO_2_ films^47,48^. Another reason for the higher optical transmittance of the templated films can be related to less root-mean-square (RMS) roughness of the surface of titania CT-TiO_2_ films prepared via the dip-coating method^47^. Therefore, increasing the light transmittance using P123 templates increases the light-harvesting, which helps improve the efficiency of the cells.

**Figure 2:**
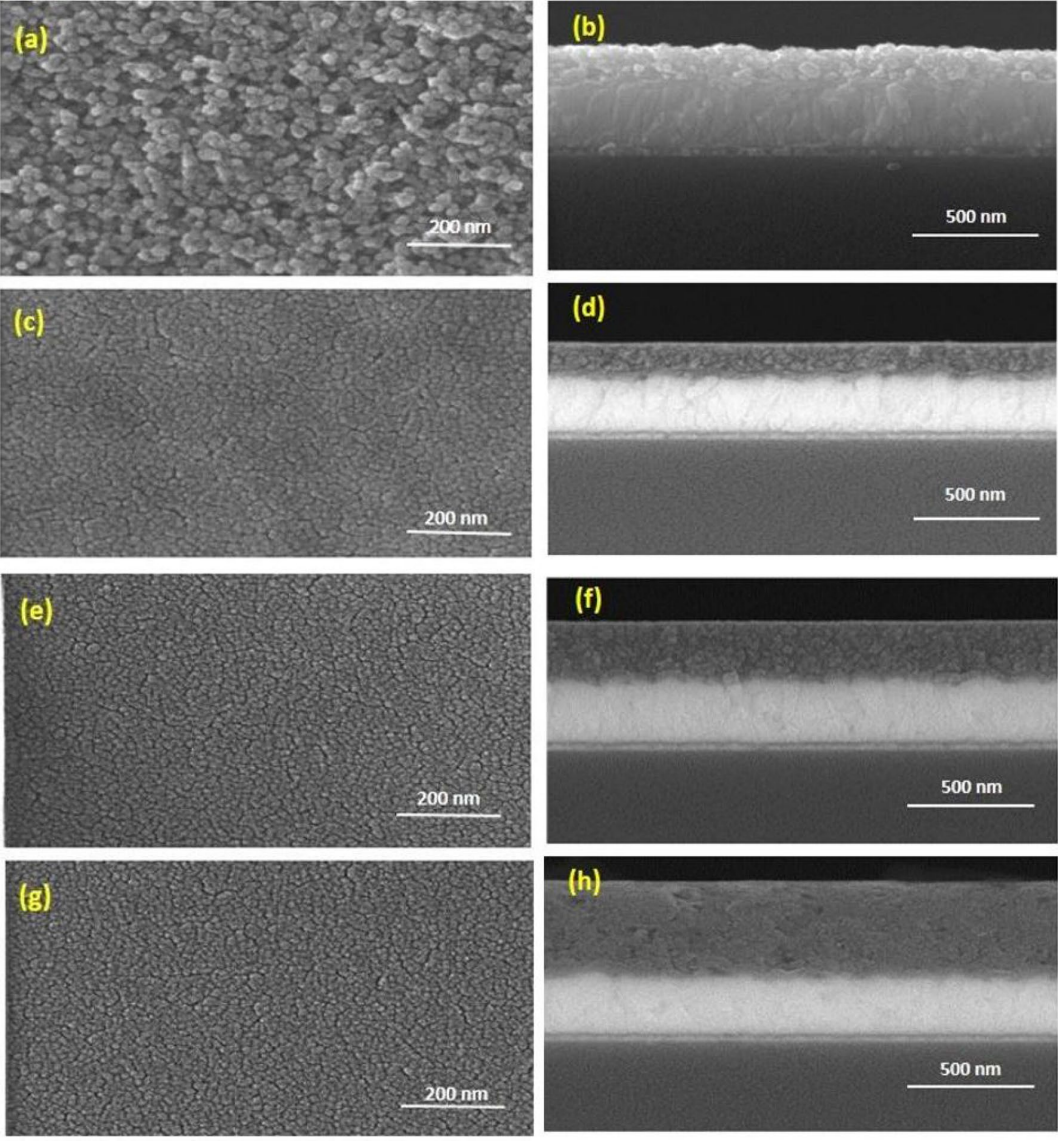
Top view and cross-sectional FE-SEM images of NC-TiO_2_ and CT-TiO_2_ layers on FTO coated glass (~ 15 Ω/sq) (**a**,**b**) RP-TiO_2_-1 layer, (**c**,**d**) CT-TiO_2_-1 layer, (**e**,**f**) CT-TiO_2_-2 layer, and (**g**,**h**) CT-TiO_2_-3 layer (4, 5, and 6 CT-TiO_2_ layers are shown in the supporting information).

Atomic force microscopy (AFM) was performed to investigate the effect of root-mean-square (RMS) roughness on input light transmittance. The AFM images indicate that the RMS of the surface for the 1 to 3 layers of CT-TiO_2_ and blank FTO (15 Ω/sq) film were 22.5, 29.9, 36.3, and 37 nm, respectively (Table 1 and Fig. 1c). As observed, the 1-layer CT-TiO_2_ film surface is the smoothest, and all CT-TiO_2_ films are smoother than blank FTO glass. Overall, as UV–Vis spectra show, lower RMS values can increase the input light transmittance. Meanwhile, the lower the surface roughness, the more input light passes through this layer to reach the perovskite active layer. On the other hand, with the decrease in roughness, the surface becomes smoother. Furthermore, FE-SEM images were used to observe the morphology of RP-TiO_2_ and CT-TiO_2_ films with different thicknesses and discover the correlation between thickness and connections between particles (Fig. 2).

**Table 1:** Relationship between layers and roughness.

Number of layers	Roughness (nm)
(a) 1 layer	22.5
(b) 2 layer	29.9
(c) 3 layer	36.3
(d) Blank FTO (15 Ω/sq)	37

**Figure 3:**
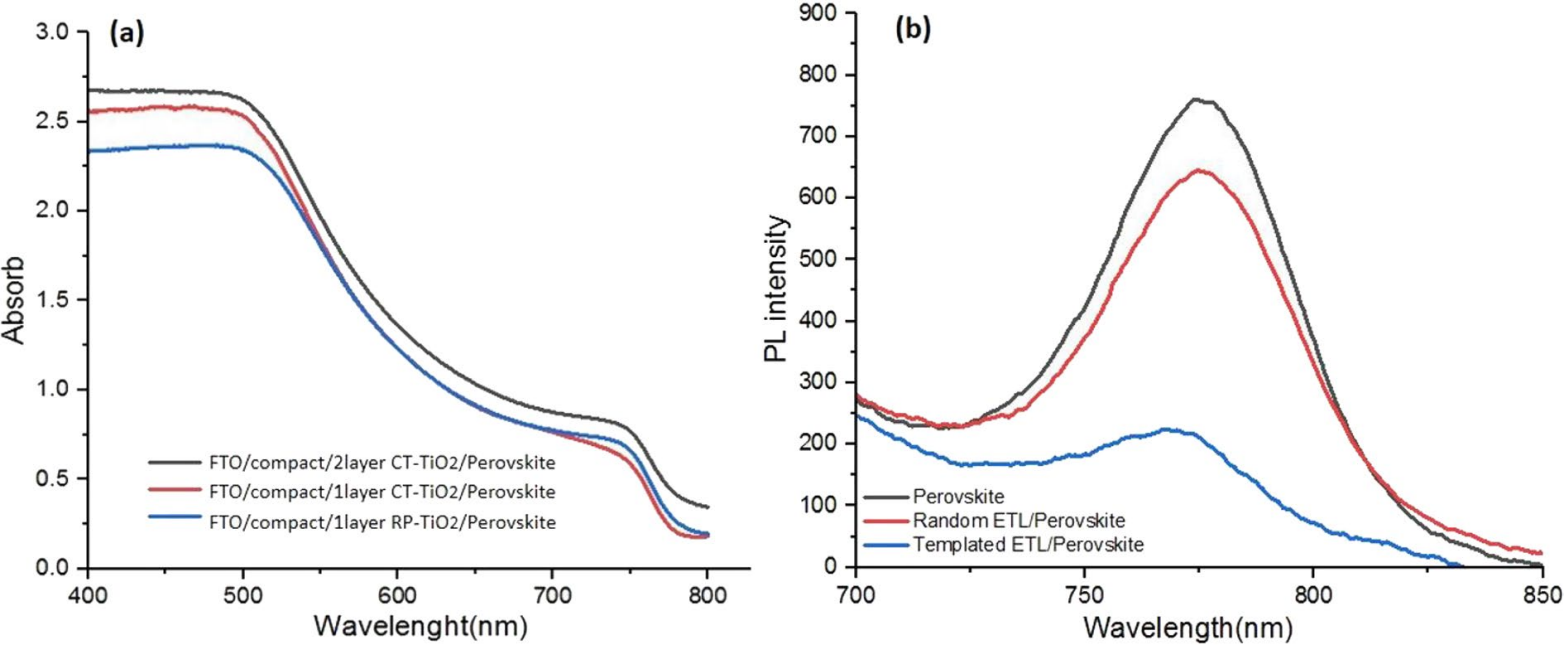
(**a**) The UV–Vis absorption spectra of the perovskite on different titania films, and (**b**) PL intensity for perovskite, RP-TiO_2_ ETL/perovskite, and CT-TiO_2_ ETL/perovskite.

As observed, unlike the RP-TiO_2_ layer, all the CT films are uniform and smooth. Similar results were observed in the literature^49^. Moreover, the particle sizes of the CT-TiO_2_ films are very small compared to the RP-TiO_2_. On the other hand, the CT-TiO_2_-1 layer and CT-TiO_2_-2 layer are pinhole free. However, with increasing the thickness to 3 layers due to repeated deposition and calcinations, significant pinholes between the titania particles is appeared, and the connections between the particles are reduced. Reducing the connection in the arrangement of electron transfer particles can make electron transport more difficult and reduce the photocurrent. Finally, it is worth mentioning that FE-SEM images confirm AFM results, especially the smoothness of the CT-TiO_2_ layers. Thus the perovskite layer could be better placed on the CT electron transfer layer, which causes fewer trap paths and consequently decreased recombination^50–52^. This can be confirmed by UV–Vis absorption spectroscopy and photoluminescence (PL) of the perovskite on the CT and RP ETL (Fig. 3).

To study the charge extraction from the perovskite layer to CT and PR TiO_2_ ETLs, photoluminescence (PL) spectroscopy was carried out. As a result, the PL intensity of CT-TiO_2_ ETL/perovskites is weaker than that of the RP-TiO_2_ ETL/perovskite configuration. This confirms that the electron transfer from perovskite to CT-TiO_2_ is greater than that of the RP-TiO_2_ structure, which in turn shows better interconnection on the interface of CT-TiO_2_/perovskite compared to RP-TiO_2_/perovskite.

### Photovoltaic performances of titania templated electron transport layer (ETL)

**Figure 4:**
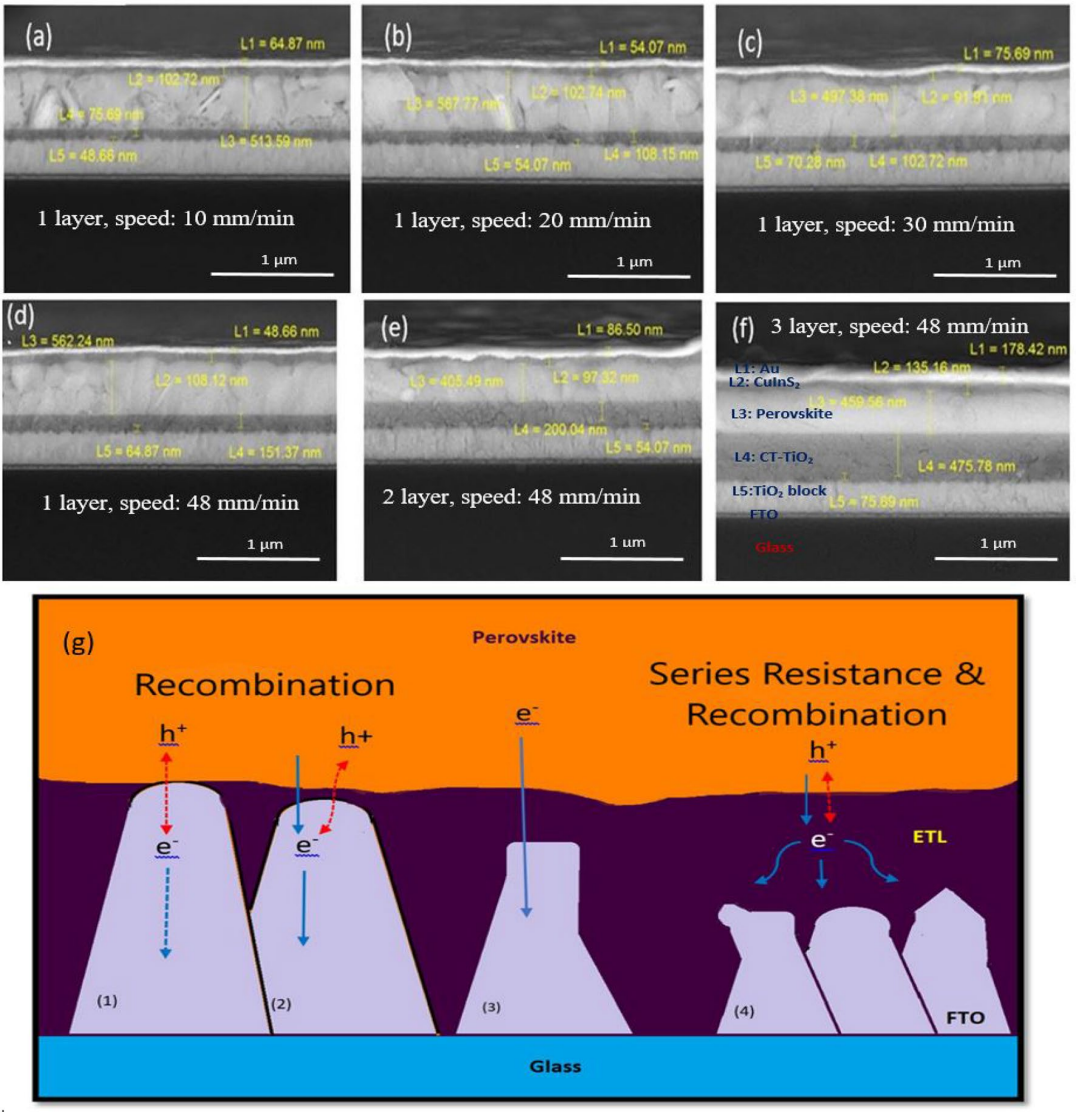
(**a**–**f**) Cross-sectional FE-SEM images of perovskite devices with different thicknesses of ETL including a configuration of FTO/TiO_2_ block layer/TiO_2_ layer/Cs_0.05_(MA_0.17_FA_0.83_)_0.95_Pb(I_0.83_Br_0.17_)_3_ /CIS/Au. (**g**) Electron transfer and electron transport mechanisms in different thicknesses of ETL. (1) No ETL (e^−^ (FTO) + h^+^(perovskite)), (2) ultra-thin ETL, (3) ideal thickness of ETL (best performance), (4) ultra-thick ETL, and (e^−^ (ETL) + h^+^ (perovskite)).

A titania templated film prepared by P123 block copolymer was used as ETL to substitute conventional PR-TiO_2_ meso layer in CIS-PSCs. In this regard, the ETL thickness was changed by the number of layers and the change in the withdrawal speed of dip-coating. The cross-sectional FE-SEM images of perovskite devices with a configuration of FTO/compact TiO_2_/CT-TiO_2_/Cs_0.05_(MA_0.17_FA_0.83_)_0.95_Pb (I_0.83_Br_0.17_)_3_/CIS/Au have been demonstrated in Fig. 4.

The cross-sectional FE-SEM images of perovskite devices with different thicknesses of ETL utilizing the dip-coating method indicate that different thicknesses of mesoporous templated films have been obtained by changing the number of layers and withdrawal deposition speeds.

**Figure 5:**
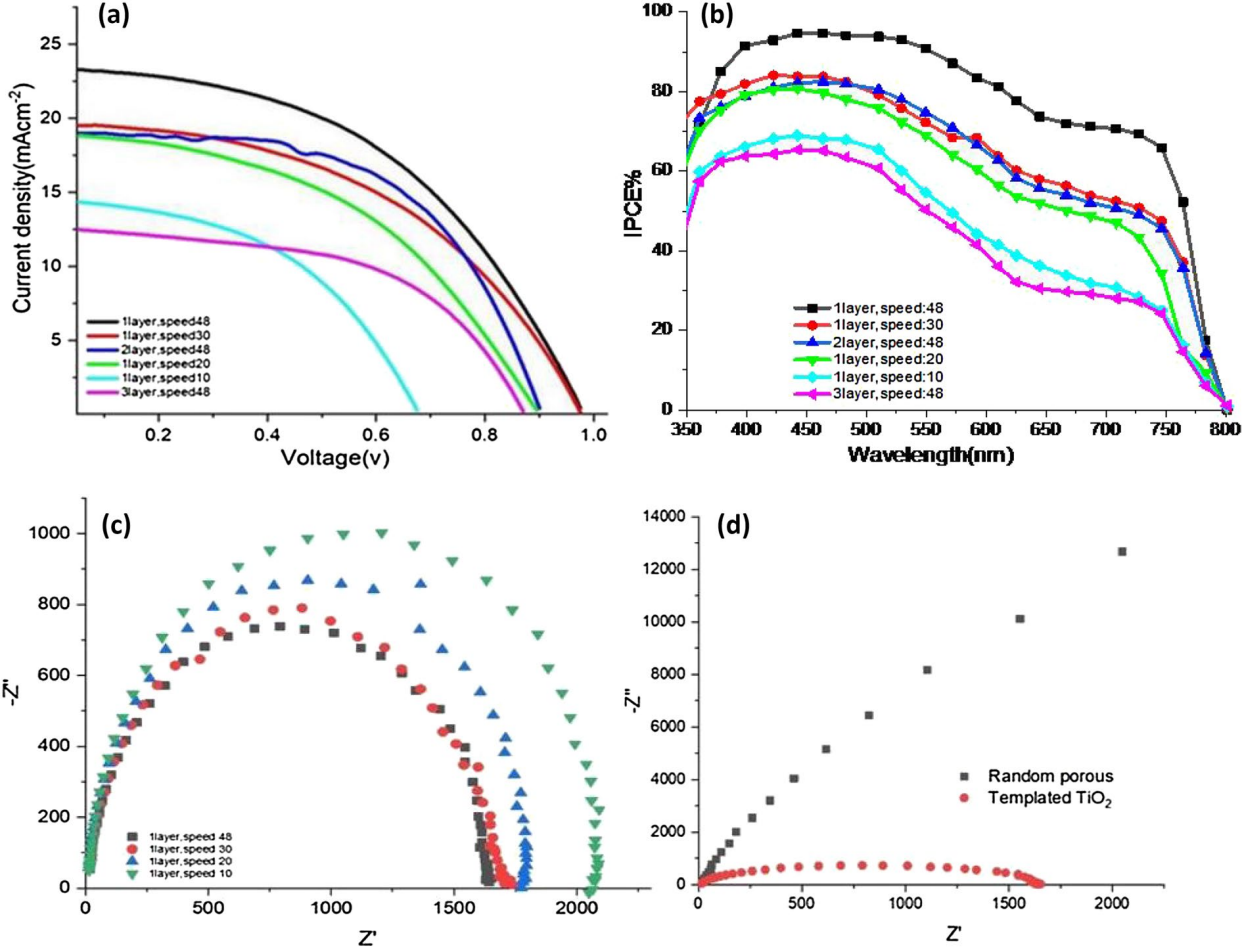
(**a**) *J–V* curve of PSCs bearing different thicknesses of CT-TiO_2_. (**b**) IPCE analysis of CIS-PSCs devices with different thicknesses of CT-TiO_2_ ETL. (**c**) Nyquist plots of CIS-PSCs devices with different thicknesses of 1 layer of CT-TiO_2_ ETL prepared by different withdrawal dip-coating speeds. (**d**) Compared impedance Nyquist of random porous and best templated porous.

Table 2 and Fig. 5a illustrate the photocurrent density to voltage (*J–V*) characteristics of CIS-PSCs bearing various thicknesses of CT-TiO_2_ films.

**Table 2:** Effect of the thickness of CT and RP-TiO_2_ layer on the device performance of CIS-PSCs (the active area of the cells is 0.09 cm^2^).

	Layer thickness (nm)	*J*_SC_ (mA cm^−2^)	*V*_oc_ (V)	Fill factor	Efficiency (%)
1-layer, speed: 10	75.69	14.41	0.655	0.446	4.21
1-layer, speed: 20	108.15	18.68	0.855	0.42	6.71
1-layer, speed: 30	118.93	19.51	0.905	0.466	8.22
1-layer, speed: 48	151.37	23.35	0.995	0.477	11.08
2-layer, speed: 48	200.04	18.97	0.824	0.518	8.09
3-layer, speed: 48	475.78	12.55	0.885	0.466	5.17
1-layer RP	145.97	20.52	0.965	0.637	12.61

As observed in the table, by increasing thickness up to 151.37 nm, *J*sc, *V*oc, and fill factor have increased while decreasing at higher thicknesses. Thus, maximum *Jsc*, *V*oc, and fill factor were observed for the CIS-PSC with a thickness of 151.37 nm (image 4d). The effect of ETL thickness on electron extraction and transporting is significant because thinner ETL increases the risk of pinholes directly contacting the FTO substrate and perovskite layer. Moreover, the high thickness of ETL gives rise to an increment in series resistance and electron–hole recombination. As a result, in both cases, the Jsc and FF of the devices decrease^53,54^. Thus, achieving the optimum thickness for ETL is very important (Fig. 4g).

As observed in Fig. 5, one layer of templated ETL deposited at the speed of 48 mm/min and the thickness of 151.37 nm showed the highest *J*_sc_ and best performance. According to the transmittance and absorption diagram in Figs. 1b and 3a, the higher the transmitted light, the higher the absorption with perovskite and current efficiency^40^. However, increasing the thicknesses beyond 151.37 nm can raise series resistance and electron–hole recombination. The Incident Photon to Current Efficiency (IPCE) curves of the CIS-PSCs measured in wavelength light absorption range of 400–800 nm are shown in Fig. 5b. These IPCE data are in line with the *J–V* curves. Regarding the *Voc* data, by reducing the band gap (Fig. S7), more electrons diffuse from the valence band to the conduction band, which results in an increase in open circuit voltages. The closer the Fermi level to the conduction band edge, the better electron extraction to the bottom electrode^49^.

In the next step, the photovoltaic performance of the best cell, which was TiO_2_ templated single layer prepared at 48 mm/min, dip-coating speed, was compared with that of the standard cell. The current density in the CT-TiO_2_ is higher than that of the standard cell because the former has a less refractive index, which helps more light enter the cell and increases light absorption by perovskite materials. However, the fill factor, which indicates the arrangement of particles and pores and is essential for electron transport to the external circuit, was lower than that of the standard cell. Notably, FF in the CT-TiO_2_ cell increases during 90 days of the stability test discussed in the stability part. Figure 5c shows the impedance spectra (Nyquist plots) of CIS-PSCs. Under optimum conditions (1 layer, speed 48), the device exhibits the lowest resistance (diameter of the arc). The optimization of processing conditions leads to minimum transport resistance within the CT-TiO_2_ films. The Nyquist plot for CIS-PSCs-based RP-TiO_2_ compared to the best CT-TiO_2_ films is shown in Fig. 5d. Also, the Nyquist plot for CIS-PSCs-based RP-TiO_2_ is shown in Fig. S2.

### Stability of titania templated electron transport layer in CIS-PSCs

**Figure 6:**
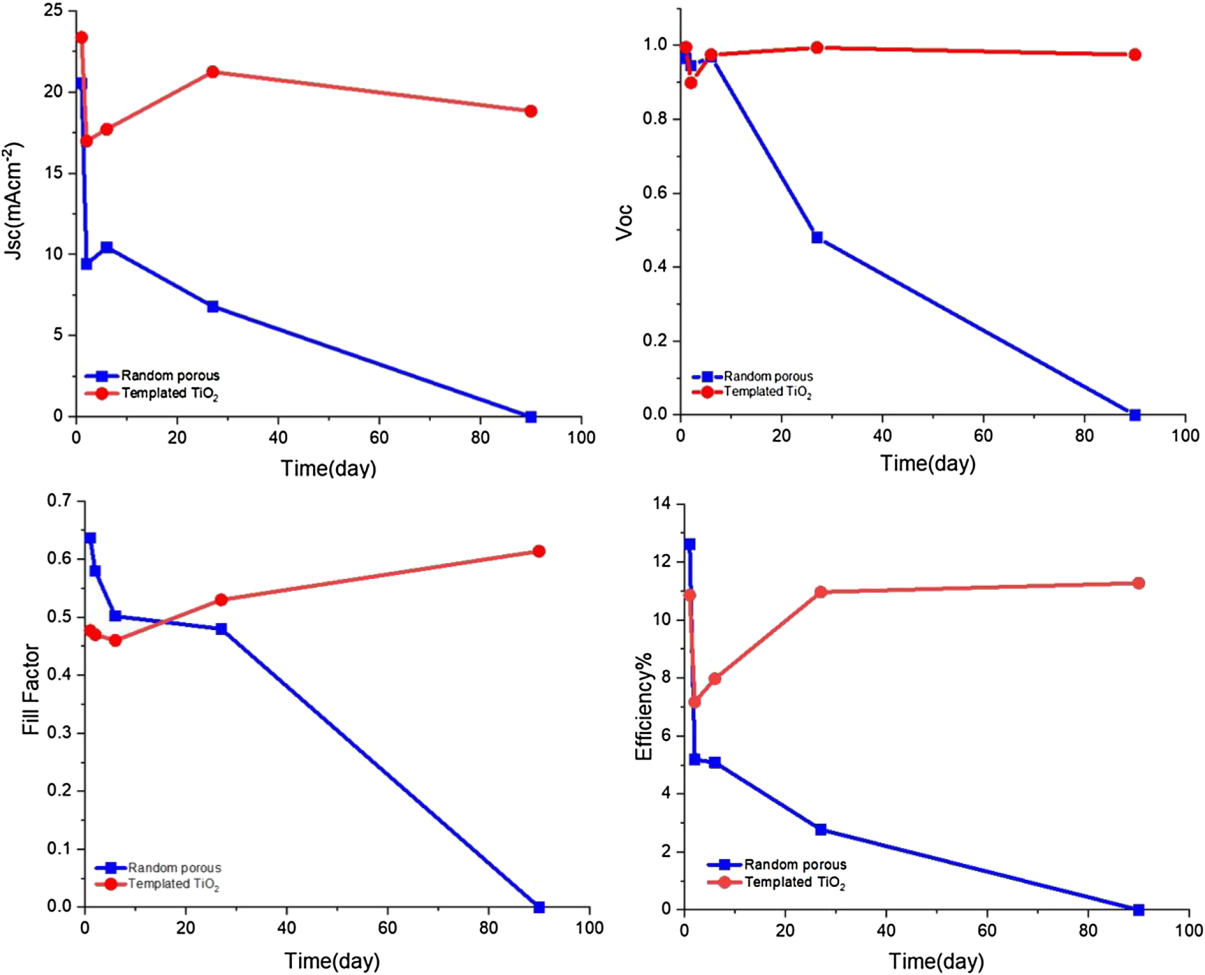
Stability of photovoltaic parameters of standard and templated TiO_2_-based CIS-PSCs measured during 90 days under ambient conditions (25–30 °C, relative humidity of 30–45%). Evolution of *J*_sc_, *V*_oc_, FF, and efficiency.

The CIS-PSCs with CT-TiO_2_ (thickness: 151.37 nm) and RP-TiO_2_ ETLs were compared in stability tests for 90 days at ambient temperature and humidity (Table 3 and Fig. 6).

**Table 3:** PV performance of CIS-PSCs with CT and RP TiO_2_ ETLs measured during 90 days at ambient temperature and humidity.

Cell	*J*sc CT	*J*sc RP	*V*oc CT	*V*oc RP	FF CT	FF RP	η CT	η RP
First day	23.35	20.52	0.995	0.960	0.477	0.637	11.08	12.61
Second day	16.97	9.40	0.899	0.945	0.470	0.580	7.17	5.18
Sixth day	17.70	10.44	0.975	0.970	0.460	0.502	7.94	5.09
Twenty-seventh day	21.23	6.80	0.974	0.850	0.530	0.480	10.96	2.77
Ninetieth day	18.82	0	0.975	0	0.614	0	11.27	0

As shown in Fig. 6, on the first day, the random porous cell (standard cell) presents a higher fill factor and, therefore, higher efficiency than the templated cell. However, the FF in the templated cell improves over time. Interestingly, the power conversion efficiency of the templated CIS-PSCs enhanced from 11.08 to 11.27% after 90 days. This can probably be due to the completion of pore filling in templated TiO_2_ during the time. In other words, first, the perovskite materials do not probably fill the pores of the CT-TiO_2_ electron transfer layer well and reduce the FF compared to the RP-TiO_2_ cells. Then, over time, the perovskite materials fill the templated pores, increasing FF and efficiency^55–57^. Moreover, the reduction of defects in CT-TiO_2_ after exposure to water molecules and recovery can be another reason for increasing efficiency.

It is worth mentioning that the templated CIS-PSCs retain their initial efficiencies at ambient conditions over 90 days without encapsulation. In contrast, the efficiency of control devices based on RP-TiO_2_ drops rapidly, reaching zero under the same aging conditions.

$$ {\text{CH}}_{3} {\text{NH}}_{3} {\text{PbX}}_{3} \;({\text{s}})\mathop{\longrightarrow}\limits^{{{\text{H}}_{2} {\text{O}}}}{\text{CH}}_{3} {\text{NH}}_{3} {\text{X}}\;({\text{s}}) + {\text{PbX}}_{2} \;({\text{s}}) $$

**Figure 7:**
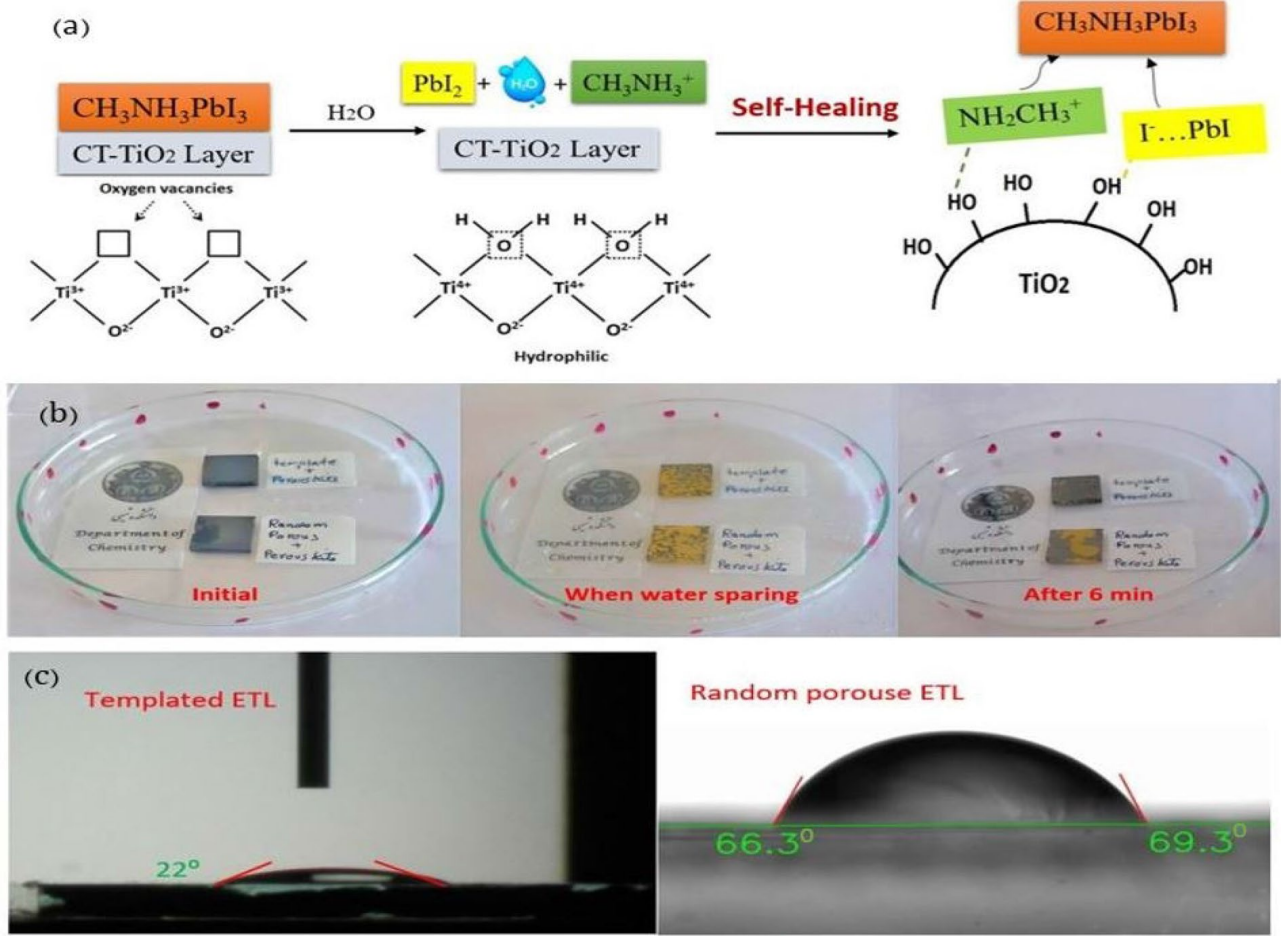
(**a**) Schematic representation of the mechanism for the self-healing perovskite on surface templated TiO_2_. (**b**) Images of perovskite films in healing test in the beginning, upon water-spraying, and after 6 min (movies and other images are in the supporting information), and (**c**) contact angle images of water on the templated structure (22°) and random porous (67°) TiO_2_ structure.

As previously indicated, lead halide perovskite materials are susceptible to ambient humidity and easy to dissolve and degrade in a humid environment, as shown in the following reaction^58,59^. This irreversible reaction can be reversed by self-healing with the help of the surface –OH groups and hydrogen bonding. The –OH groups of the templated TiO_2_ surface can be bonded to the nitrogen of the methylammonium on one side and lead halide on the other, which helps bring methylammonium and lead halide closer and regenerate the perovskite composition^57^. Having been kept away from water vapor, PbI_2_ in the film reacts with nearby MAI to regenerate the perovskite MAPbI_3_ phase, which is very similar to the two-step synthesis. The instant decomposition-regeneration mechanism explains the fast self-healing process. The corresponding mechanism for the self-healing of perovskite on the surface of the templated TiO_2_ is observed in Fig. 7a,b, which show the images of perovskite films in healing tests. As observed in the figures, the perovskite film and the corresponding devices can recover rapidly to their original phase and performance after vapor spraying and drying in ambient air.

To evaluate the water-repellency properties of ETL surfaces, water contact angle measurements were performed on the surfaces of the CT-TiO_2_ and RP-TiO_2_. The CT ETL contact angle is 22, much lower than RP-TiO_2_ (67). This shows that there are much more OH groups on the surfaces of the templated TiO_2_ compared with random TiO_2_, which can improve perovskite self-healing more effectively in the presence of moisture (Fig. 7c).

**Figure 8:**
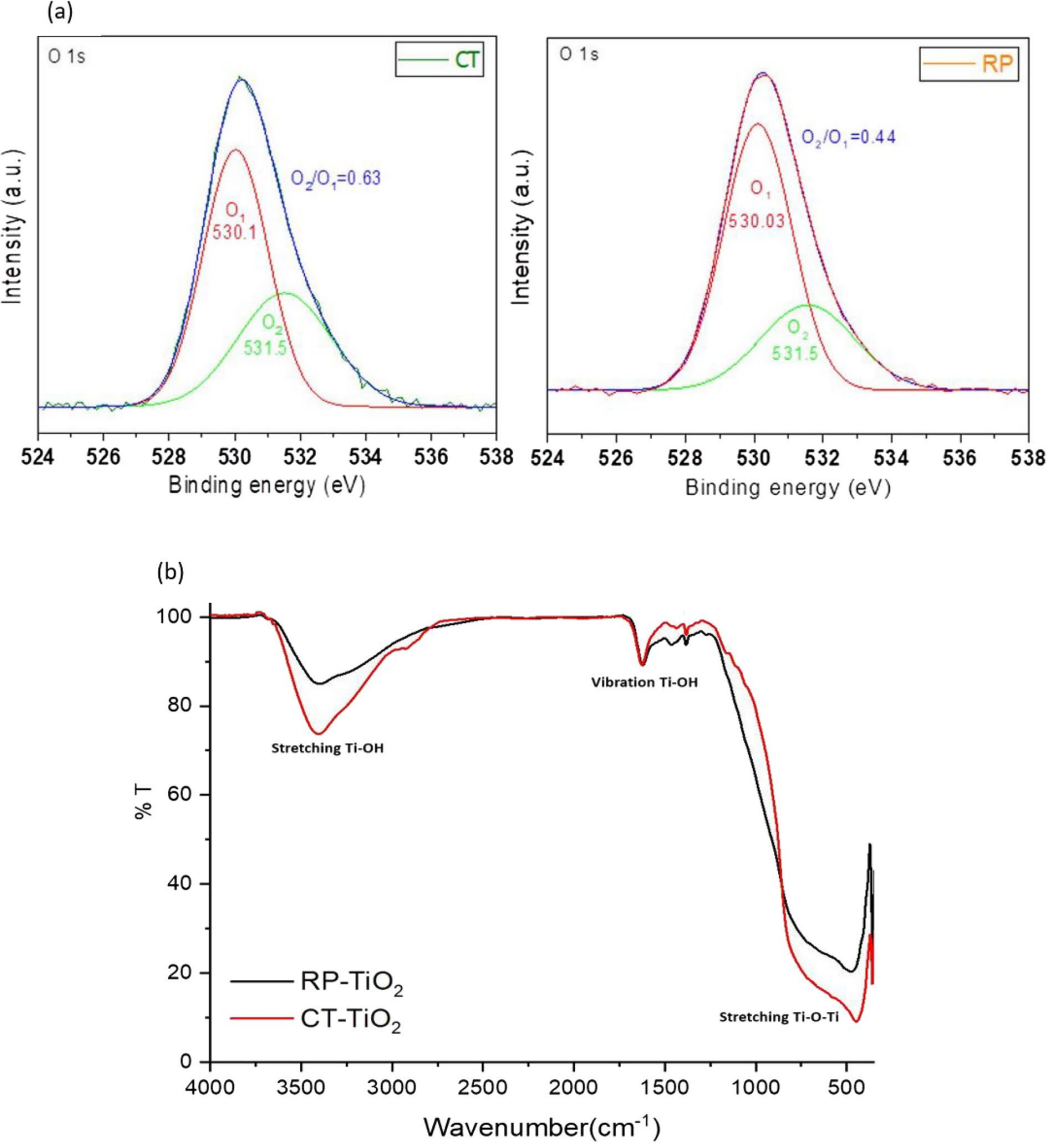
(**a**) High-resolution O 1 s XPS spectra and (**b**) FT-IR spectra of CT-TiO_2_ and RP-TiO_2_.

Based on the high-resolution XPS spectrum of the O 1 s shown in Fig. 8a, two peaks are observed, with the O1 peak centered at ≈ 530.1 eV attributed to the lattice oxygen and the O_2_ peak centered at ≈ 531.5 eV to the surface adsorbed OH groups. Comparing the two samples, the CT-TiO_2_ sample has a higher ratio of O2/O1, suggesting a higher hydroxyl group concentration. In other words, the oxygen vacancies in the templated film are prone to adsorb H_2_O molecules after exposure to water. The water molecules on the surface of the titania film are adsorbed in the oxygen vacancy, forming chemisorbed H_2_O (surface hydroxyl). Thus, these –OH groups may be responsible for the self-healing property of the templated film^60–62^. Meanwhile, the XPS spectra for the samples before facing the water molecules are shown in Fig. S6.

The FTIR spectra of TiO_2_ samples are shown in Fig. 8b. The broad band centered at 3700–2700 cm^−1^ is assigned to the intermolecular interaction of the hydroxyl group for water molecules with the TiO_2_ surface. As observed, the peak area for CT-TiO_2_ increased, confirming that CT-TiO_2_ encompasses more surface OH groups compared to the RP-TiO_2_. The broad band centered at 500–600 cm^−1^ is attributed to the bending stretching (Ti–O–Ti) bonds in the TiO_2_ lattice. It appears that, after the H_2_O molecules are trapped by oxygen vacancies in CT-TiO_2_, bridging oxygen is formed^63^. This, in turn, can be a reason for improving the performance of the CT-TiO_2_ device over time after exposure to water molecules.

Furthermore, the stabilized efficiency and photocurrent density of CIS-PSCs biased at maximum power points and also the efficiency of CIS-PSC-based CT-TiO_2_ and RP-TiO_2_ under light-soaking are shown in Figs. S8 and S9. As can be seen, the photocurrent of the CT-TiO_2_-based device quickly reached the steady-state photocurrent. However, the photocurrent of the RP-TiO_2_-based cell needed more time to saturate, which is consistent with the larger hysteresis of the device based on RP-TiO_2_. Considering the light-soaking profile, the RP-TiO_2_-based cell exhibited more severe degradation compared to the CT-TiO_2_ device. This can be related to the suppressed activation of the trap sites originating from the oxygen vacancies of the CT-TiO_2_ layer. This may be also attributed to the higher UV stability of the CT-TiO_2_-based cell due to a smaller anatase crystallite size compared with the RP-TiO_2_^64^.

## Conclusion

To improve the photocurrent density and efficiency of low-efficient copper indium disulfide-based PSCs, uniform copolymer-templated TiO_2_ (CT-TiO_2_) ETLs (1, 2, and 3 layers with different dip coating rates) were employed as alternatives for conventional random porous TiO_2_ ETL. XRD results confirmed an anatase TiO_2_ structure. CT-TiO_2_ ETLs enhanced the input light transmittance into the cell owing to its lower refractive index and therefore raised the photocurrent density and photovoltaic performance. Meanwhile, 1-layer CT-TiO_2_ ETLs prepared with a dip coating rate of 48 mm min^−1^ presented the highest performance in the PSC device. Interestingly, CT-TiO_2_ induced a self-healing effect in perovskite, probably due to a large number of surface hydroxyl groups on TiO_2,_ and therefore presented superior stability in CIS-PSC. In this regard, CIS-PSC-based 1-layer CT-TiO_2_ exhibited a conversion efficiency of 11.27% and a high photocurrent density of approximately 23 mA/cm^2^. Moreover, these unsealed CIS-PSCs retained their performance in stability tests during 90 days at ambient conditions. Considering all the factors mentioned, templated TiO_2_ layers with more oxygen vacancies have great potential as highly efficient and smoothly fabricated ETLs for inducing self-healing properties in unstable perovskite materials of PSCs.

## Experimental section

### Materials

Titanium isopropoxide (Ti(IP)_4_, 95%), Zn powder (particle size $<45\, \upmu$m), Hydrochloric acid (HCl, 36 wt%), Ethanol ($\ge$ 99.99%), Acetone ($\ge 99.5\%$), 2-propanol ($\ge 99.8\%$), N,N-Dimethylformamide (DMF, 99.8%), Dimethyl sulfoxide (DMSO, 99.8%) were purchased from Merck. Cesium iodide (CsI, 99.9%), Pluronic P123 surfactant, 1-butanol (99.8%), Chlorobenzene (99.98%), Chloroform ($\ge 99\%)$ were obtained from Sigma-Aldrich. Lead bromide (PbBr_2_, 99.99%), Lead iodide (PbI_2_,99.99%), Formamidinium iodide (FAI, $>99.5\%$), Methyl ammonium bromide (MABr, $>99.5\%$) were supplied from Luminescence technology corp (Lumtec). TiO_2_ paste (PST-20T) and CuInS_2_ ink (INK-20 CIS) were prepared from Sharif Solar. FTO (resistance 15 Ω sq^−1^) coated glass was supplied from Dyesol. Gold (Switzerland, 99.99%). All chemicals were used without further purification.

### Preparation of initial solutions for coating

**Figure 9:**
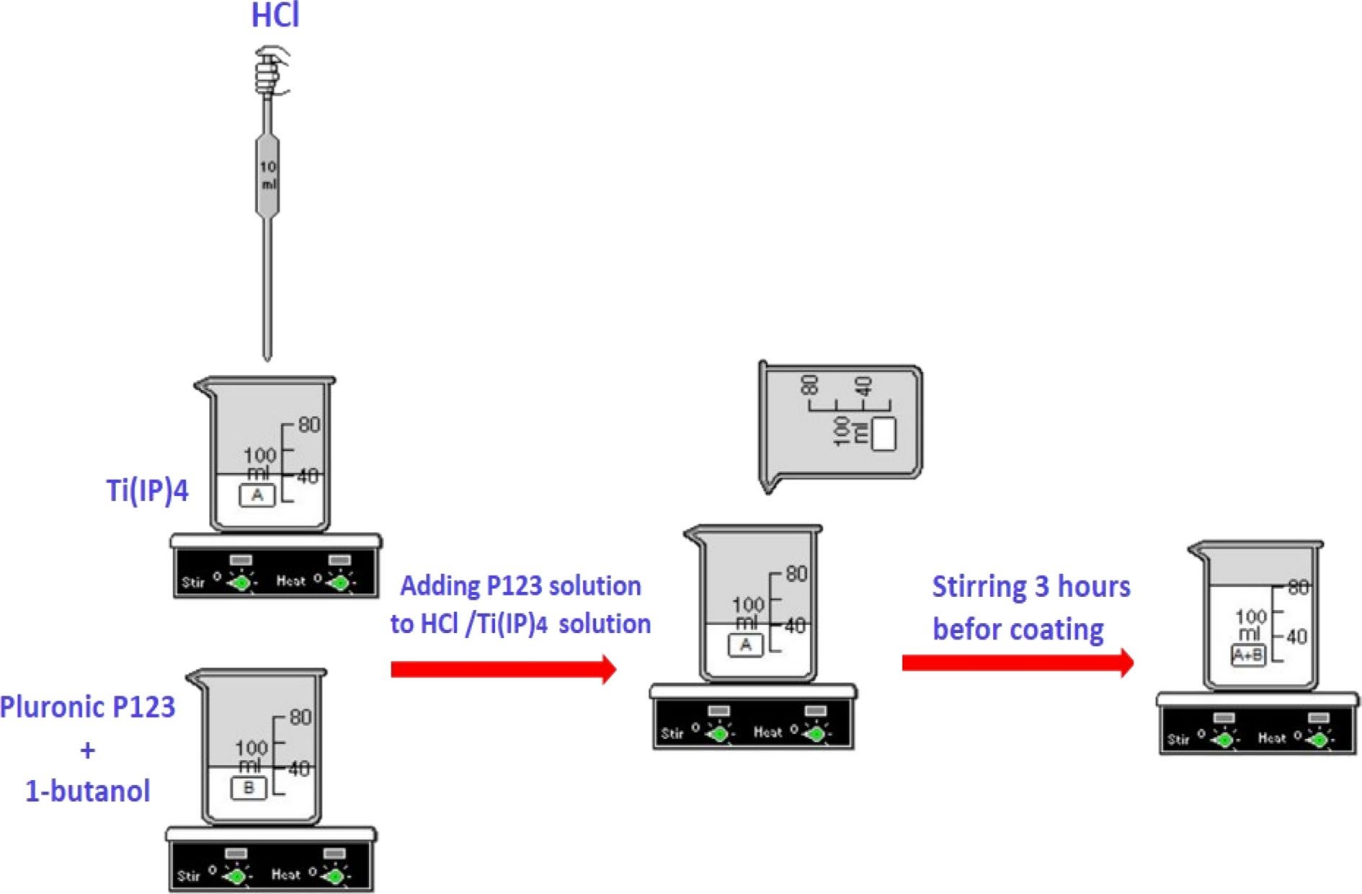
Scheme of the preparation procedure for CT-Solution.

To prepare the solution containing P123 copolymer^34–36^, first, P123 copolymer (2 g) was dissolved in three different quantities of 1-butanol (Aldrich, 18.15, 27.22, and 36.3 g). These three mother solutions in various concentrations were then vigorously stirred to dissolve the P123 copolymer completely. Next, concentrated HCl (Merck, 36% wt, 4.85 g) was slowly added to titanium tetraisopropoxide (Merck, 6.35 g) under vigorous stirring on an ice bath due to the exothermic nature of the hydrolysis and condensation reactions. Finally, the solutions of P123 were slowly added to the HCl/Ti(IP)_4_ solutions, followed by stirring for 3 h before coating (Fig. 9).

### Fabrication of perovskite solar cells

To electrically isolate the photoanode and cathode, etching of FTO substrates was performed by zinc powder and diluted HCl^40^. The electrodes were then cleaned by sonication in ethanol, acetone, DI water, and isopropanol for 15 min, respectively. Next, to remove the traces of organic residues, the substrates were heated at 450 °C for 30 min. Next, a TiO_2_ solution containing TTIP in anhydrous ethanol (0.15 M) was spin-coated and then annealed at 500 °C to deposit a compact layer on the FTO substrate. Next, to coat the random porous TiO_2_ layer, a commercial TiO_2_ NPs paste was diluted in ethanol solvent (1:5.5 wt. ratio) and coated on FTO/compact TiO_2_ films by spin-coating at 4000 rpm for 30 s. Finally, to remove terpineol and cellulose and form the titania anatase phase, the porous layer was annealed at 500 °C for 1 h.

For templated mesopores, TiO_2_ film was prepared by dip-coating (withdrawal rates of 48, 30, 20, and 10 mm/min) of the solution on TiO_2_ compact coated on FTO. Next, several layers were deposited via a stabilization step between each coating cycle. This stabilization process was executed by heating the fresh film for 15 min on a hot plate pre-heated at 300 °C. Afterward, the final sintering of the prepared films was conducted at 450 °C for 1 h (heating rate: 1 °C/min) to fully condense the inorganic network, crystallize the anatase phase and remove all surfactant residues.

**Figure 10:**
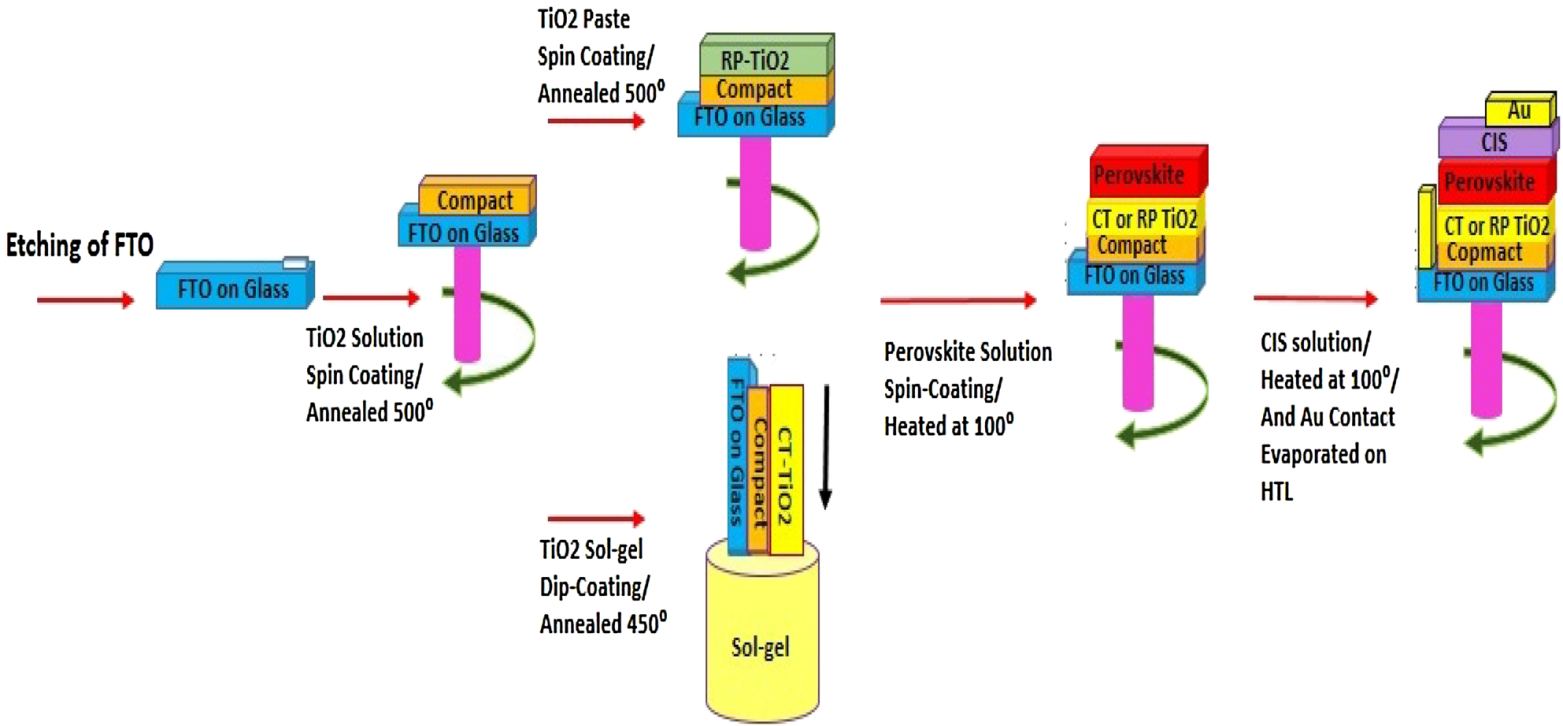
Schematic illustration of the PSC fabrication based on CT and RP ETLs.

First, to coat the Cs_0.05_(MA_0.17_FA_0.83_)_0.95_Pb(I_0.83_Br_0.17_)_3_ perovskite, a solution containing FAI (1 M), CsI (0.05 M), PbI_2_ (1.1 M), MABr (0.2 M), and PbBr_2_ (0.22 M) in anhydrous DMF: DMSO solvent (4:1 volume ratio) was prepared, which was then deposited on TiO_2_ using spin-coating method via a two-step process at 1000 rpm, 10 s, and 4000 rpm for 20 s. Chlorobenzene solvent was slowly added during the final 5 s of the second step of the spin coating process. The as-prepared layers were then heated at 100 °C for 1 h. Next, the inorganic HTL CIS layer was prepared according to a reported method^31^. In a typical method, a CIS solution in chloroform (20 mg/mL) was spin-coated over the perovskite film at 3000 rpm for 60 s and then heated at 100 °C for 5 min. Finally, the metal contact (Au) was evaporated onto the HTL (Fig. 10).

### Characterization

X-ray diffraction (XRD, Bruker D8 advance, Cu Kα, λ = 1.54 Å, scan rate of 0.04 (2θ/s)) was used to characterize the crystalline phase. Field emission scanning electron microscopy (FE-SEM, TESCAN, Czech Republic) and atomic force microscopy (AFM, Dualscope/Rasterscope C26, DME, Denmark) were used to study the morphology and thickness of the prepared films. The optical characteristics of the films were studied by UV–Vis spectroscopy using a Cary instrument (wavelength range of 190–900 nm). The solar cells' photocurrent–voltage (J–V) characteristics were obtained under one sun (AM1.5G, 100 mW/cm^2^) light illumination using Sharif solar simulator. Electrochemical impedance spectra (EIS) were recorded in the dark under a reverse bias of V = 0 to Voc. The frequency range was set from 100 to 2 × 10^5^ Hz. The EIS measurements were carried out using an AUTOLAB PGE-18 (Metrohm Autolab, Utrecht, and The Netherlands) potentiostat/galvanostat. The incident photon to current conversion efficiency (IPCE) was measured using Sharif Solar IPCE-015 equipment. Using an Avaspec2048Tech spectrophotometer, the steady-state photoluminescence (PL) spectra were recorded. The FTIR spectra were recorded on Fourier Transform Infrared Spectroscopy (FT-IR) 6300 JASCO, Japan. X-ray Photoelectron Spectroscopy (XPS) UHV analysis system, SPECS Company, was used to determine the surface concentration of hydroxyl groups.

## Supplementary Information

Supplementary Legends.

Supplementary Video 1.

Supplementary Video 2.

Supplementary Information.

## Data Availability

All data generated or analysed during this study are included in this published article and its supplementary information files.

## References

[1] Hodes G (2013). Perovskite-based solar cells. Science.

[2] Liu M, Johnston MB, Snaith HJ (2013). Efficient planar heterojunction perovskite solar cells by vapour deposition. Nature.

[3] Burschka J (2013). Sequential deposition as a route to high-performance perovskite-sensitized solar cells. Nature.

[4] Ball JM, Lee MM, Hey A, Snaith HJ (2013). Low-temperature processed meso-superstructured to thin-film perovskite solar cells. Energy Environ. Sci..

[5] Lee MM, Teuscher J, Miyasaka T, Murakami TN, Snaith HJ (2012). Efficient hybrid solar cells based on meso-superstructured organometal halide perovskites. Science.

[6] Kim H-S (2012). Lead iodide perovskite sensitized all-solid-state submicron thin film mesoscopic solar cell with efficiency exceeding 9%. Sci. Rep..

[7] Akin S, Sadegh F, Turan S, Sonmezoglu S (2019). Inorganic CuFeO_2_ delafossite nanoparticles as effective hole transport materials for highly efficient and long-term stable perovskite solar cells. ACS Appl. Mater. Interfaces.

[8] Min J (2015). Interface engineering of perovskite hybrid solar cells with solution-processed perylene–diimide heterojunctions toward high performance. Chem. Mater..

[9] Grätzel M (2014). The light and shade of perovskite solar cells. Nat. Mater..

[10] Xing G (2013). Long-range balanced electron-and hole-transport lengths in organic-inorganic CH_3_NH_3_PbI_3_. Science.

[11] Manser JS, Kamat PV (2014). Band filling with free charge carriers in organometal halide perovskites. Nat. Photonics.

[12] Stranks SD (2013). Electron–hole diffusion lengths exceeding 1 micrometer in an organometal trihalide perovskite absorber. Science.

[13] Dong Q (2015). Electron–hole diffusion lengths > 175 μm in solution-grown CH3NH3PbI3 single crystals. Science.

[14] Ni Z (2022). Evolution of defects during the degradation of metal halide perovskite solar cells under reverse bias and illumination. Nat. Energy.

[15] Akman E, Akin S (2021). Poly (N, N′-bis-4-butylphenyl-N, N′-bisphenyl) benzidine-based interfacial passivation strategy promoting efficiency and operational stability of perovskite solar cells in regular architecture. Adv. Mater..

[16] Christians JA, Miranda Herrera PA, Kamat PV (2015). Transformation of the excited state and photovoltaic efficiency of CH_3_NH_3_PbI_3_ perovskite upon controlled exposure to humidified air. J. Am. Chem. Soc..

[17] Niu G, Guo X, Wang L (2015). Review of recent progress in chemical stability of perovskite solar cells. J. Mater. Chem. A.

[18] Rehman W (2017). Photovoltaic mixed-cation lead mixed-halide perovskites: Links between crystallinity, photo-stability and electronic properties. Energy Environ. Sci..

[19] Saliba M (2016). Cesium-containing triple cation perovskite solar cells: Improved stability, reproducibility and high efficiency. Energy Environ. Sci..

[20] Ren J (2019). Suppressing charge recombination and ultraviolet light degradation of perovskite solar cells using silicon oxide passivation. ChemElectroChem.

[21] Sun Y, Peng J, Chen Y, Yao Y, Liang Z (2017). Triple-cation mixed-halide perovskites: Towards efficient, annealing-free and air-stable solar cells enabled by Pb (SCN) 2 additive. Sci. Rep..

[22] Duan, L. & Uddin, A. Defects and stability of perovskite solar cell: A critical analysis. *Mater. Chem. Front.* (2022).

[23] Zhou H (2014). Interface engineering of highly efficient perovskite solar cells. Science.

[24] Heo JH (2013). Efficient inorganic–organic hybrid heterojunction solar cells containing perovskite compound and polymeric hole conductors. Nat. Photonics.

[25] Qin P (2014). Inorganic hole conductor-based lead halide perovskite solar cells with 12.4% conversion efficiency. Nat. Commun..

[26] Kim G (2022). Methylammonium compensation effects in MAPbI3 perovskite solar cells for high-quality inorganic CuSCN hole transport layers. ACS Appl. Mater. Interfaces.

[27] Yang Y (2017). Ultrasound-spray deposition of multi-walled carbon nanotubes on NiO nanoparticles-embedded perovskite layers for high-performance carbon-based perovskite solar cells. Nano Energy.

[28] Zhang H, Wang H, Chen W, Jen AKY (2017). CuGaO_2_: A promising inorganic hole-transporting material for highly efficient and stable perovskite solar cells. Adv. Mater..

[29] Lv M (2015). Colloidal CuInS2 quantum dots as inorganic hole-transporting material in perovskite solar cells. ACS Appl. Mater. Interfaces.

[30] Zhang Y (2019). An inorganic hole-transport material of CuInSe_2_ for stable and efficient perovskite solar cells. Org. Electron..

[31] Xu L (2017). Solution-processed Cu (In, Ga)(S, Se) 2 nanocrystal as inorganic hole-transporting material for efficient and stable perovskite solar cells. Nanoscale Res. Lett..

[32] Wu Q (2015). Kesterite Cu_2_ZnSnS_4_ as a low-cost inorganic hole-transporting material for high-efficiency perovskite solar cells. ACS Appl. Mater. Interfaces.

[33] Kolny-Olesiak J, Weller H (2013). Synthesis and application of colloidal CuInS_2_ semiconductor nanocrystals. ACS Appl. Mater. Interfaces.

[34] Jena AK, Kulkarni A, Miyasaka T (2019). Halide perovskite photovoltaics: Background, status, and future prospects. Chem. Rev..

[35] Mazumdar S, Zhao Y, Zhang X (2021). Stability of perovskite solar cells: Degradation mechanisms and remedies. Front. Electron..

[36] So, D. Copper indium sulfide colloidal quantum dot solar cells (2016).

[37] Khorasani A (2019). Optimization of CuIn1–X Ga X S2 nanoparticles and their application in the hole-transporting layer of highly efficient and stable mixed-halide perovskite solar cells. ACS Appl. Mater. Interfaces.

[38] Huo Q (1994). Generalized synthesis of periodic surfactant/inorganic composite materials. Nature.

[39] Sanchez C, Boissiere C, Grosso D, Laberty C, Nicole L (2008). Design, synthesis, and properties of inorganic and hybrid thin films having periodically organized nanoporosity. Chem. Mater..

[40] Keshavarzi R (2018). The effect of the number of calcination steps on preparing crack free titania thick templated films for use in dye sensitized solar cells. Mater. Sci. Semicond. Process..

[41] Keshavarzi R, Mirkhani V, Moghadam M, Tangestaninejad S, Mohammadpoor-Baltork I (2015). Highly efficient dye sensitized solar cells based on ordered and disordered mesoporous titania thick templated films. J. Mater. Chem. A.

[42] Keshavarzi R, Mirkhani V, Moghadam M, Tangestaninejad S, Mohammadpoor-Baltork I (2015). Performance enhancement of dye-sensitized solar cells based on TiO_2_ thick mesoporous photoanodes by morphological manipulation. Langmuir.

[43] Li W, Wu Z, Wang J, Elzatahry AA, Zhao D (2014). A perspective on mesoporous TiO_2_ materials. Chem. Mater..

[44] Sakatani Y (2006). Optimised photocatalytic activity of grid-like mesoporous TiO_2_ films: Effect of crystallinity, pore size distribution, and pore accessibility. J. Mater. Chem..

[45] Sadegh F (2021). Copolymer-templated nickel oxide for high-efficiency mesoscopic perovskite solar cells in inverted architecture. Adv. Funct. Mater..

[46] Hou K (2005). Highly crystallized mesoporous TiO_2_ films and their applications in dye sensitized solar cells. J. Mater. Chem..

[47] Amini M (2018). From dense blocking layers to different templated films in dye sensitized and perovskite solar cells: Toward light transmittance management and efficiency enhancement. J. Mater. Chem. A.

[48] Keshavarzi R, Molabahrami N, Afzali N, Omrani M (2020). Improving efficiency and stability of carbon-based perovskite solar cells by a multifunctional triple-layer system: Antireflective, UV-protective, superhydrophobic, and self-cleaning. Solar RRL.

[49] Arshad Z (2021). Magnesium doped TiO_2_ as an efficient electron transport layer in perovskite solar cells. Case Stud. Therm. Eng..

[50] Schwenzer B (2012). Tuning the optical properties of mesoporous TiO_2_ films by nanoscale engineering. Langmuir.

[51] Kim MJ (2010). Effects of the surface roughness on optical properties of CdS thin films. Mol. Cryst. Liq. Cryst..

[52] Mahmood K, Sarwar S, Mehran MT (2017). Current status of electron transport layers in perovskite solar cells: Materials and properties. RSC Adv..

[53] Lu H, Ma Y, Gu B, Tian W, Li L (2015). Identifying the optimum thickness of electron transport layers for highly efficient perovskite planar solar cells. J. Mater. Chem. A.

[54] Li C (2015). Perovskite solar cell using a two-dimensional titania nanosheet thin film as the compact layer. ACS Appl. Mater. Interfaces.

[55] Schmidt-Mende L, Grätzel M (2006). TiO_2_ pore-filling and its effect on the efficiency of solid-state dye-sensitized solar cells. Thin Solid Films.

[56] Leijtens T, Lauber B, Eperon GE, Stranks SD, Snaith HJ (2014). The importance of perovskite pore filling in organometal mixed halide sensitized TiO_2_-based solar cells. J. Phys. Chem. Lett..

[57] Zhao Y (2016). A polymer scaffold for self-healing perovskite solar cells. Nat. Commun..

[58] Ahn N (2015). Highly reproducible perovskite solar cells with average efficiency of 18.3% and best efficiency of 19.7% fabricated via Lewis base adduct of lead (II) iodide. J. Am. Chem. Soc..

[59] Kaltenbrunner M (2015). Flexible high power-per-weight perovskite solar cells with chromium oxide–metal contacts for improved stability in air. Nat. Mater..

[60] Guo K (2021). IOP Conference Series: Earth and Environmental Science.

[61] Banerjee S, Dionysiou DD, Pillai SC (2015). Self-cleaning applications of TiO_2_ by photo-induced hydrophilicity and photocatalysis. Appl. Catal. B.

[62] Wu C-Y, Tu K-J, Deng J-P, Lo Y-S, Wu C-H (2017). Markedly enhanced surface hydroxyl groups of TiO_2_ nanoparticles with superior water-dispersibility for photocatalysis. Materials.

[63] Vetrivel V, Rajendran K, Kalaiselvi V (2015). Synthesis and characterization of pure titanium dioxide nanoparticles by sol-gel method. Int. J. ChemTech Res..

[64] Hwang I, Baek M, Yong K (2015). Core/shell structured TiO_2_/CdS electrode to enhance the light stability of perovskite solar cells. ACS Appl. Mater. Interfaces.

