# Supplementary material for: Self-healing perovskite solar cells based on copolymer-templated TiO2 electron transport layer
Source: Sci Rep. 2023 Apr 19;13:6368. doi: 10.1038/s41598-023-33473-9 (PMC10115803; doi:10.1038/s41598-023-33473-9)
Supplement: Supplementary file 1 — Supplementary Legends. [file 41598_2023_33473_MOESM1_ESM.docx]

**Legend of videos**

1 and 2)-Self-healing property of perovskite layers on the copolymer templated TiO_2_ ETL (MP4)
