# Supplementary material for: Self-healing perovskite solar cells based on copolymer-templated TiO2 electron transport layer
Source: Sci Rep. 2023 Apr 19;13:6368. doi: 10.1038/s41598-023-33473-9 (PMC10115803; doi:10.1038/s41598-023-33473-9)
Supplement: Supplementary file 4 — Supplementary Information. [file 41598_2023_33473_MOESM4_ESM.docx]

Self-healing perovskite solar cells based on copolymer-templated TiO_2_  electron transport layer *Nakisa Lalpour,^1^ Valiollah Mirkhani,^1^* Reza Keshavarzi,^1^* Majid Moghadam,^1^ Shahram Tangestaninejad,^1^ Iraj Mohammadpoor-Baltork^1^* *and Peng Gao^2^**

^1^Department of Chemistry, Catalysis Division, University of Isfahan, Isfahan 81746-73441, Iran

*^2^CAS Key Laboratory of Design and Assembly of Functional Nanostructures, and Fujian Provincial Key Laboratory of Nanomaterials, Fujian Institute of Research on the Structure of Matter, Chinese Academy of Sciences, Fuzhou 350002, Fujian, People’s Republic of China*

**Data availability**

All data generated or analysed during this study are included in this published article and its supplementary information files.

**Supplementary Information**

Copolymer-templated TiO_2_ electron transport layers induce self-healing properties in perovskite material and excellent stability for copper indium disulfide-based PSCs


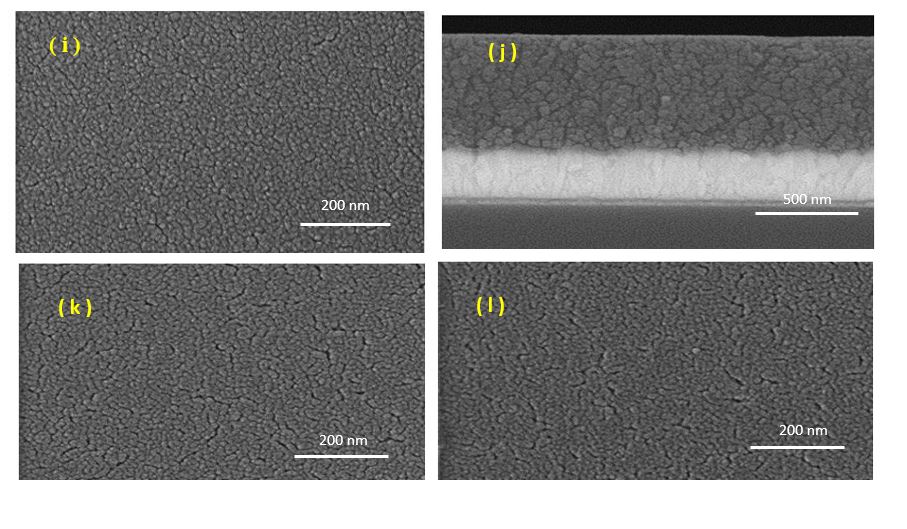


**Fig.S1**. FE-SEM images of TiO_2_ templated layers on FTO coated glass (~15Ω/sq): **(i)** Top view, **(j)** cross section of 4 layer CT-TiO_2_, **(k)** 5 layer (Top view), and **(l)** 6-layer CT-TiO_2_ (Top view).

**Fig. S2**. Impedance nyquist plot of random porous ETL in open circuit voltage

In the standard cell, at Voc, the arc opens at low frequency, this is probably related to ionic transport within the film.


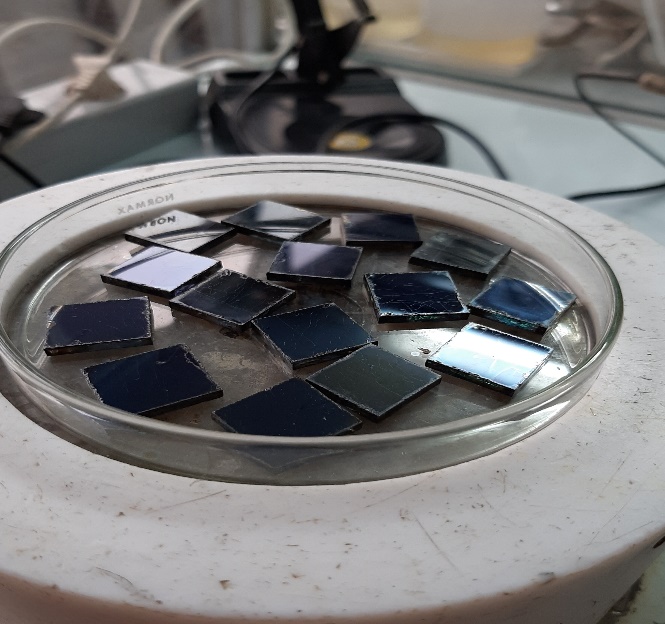

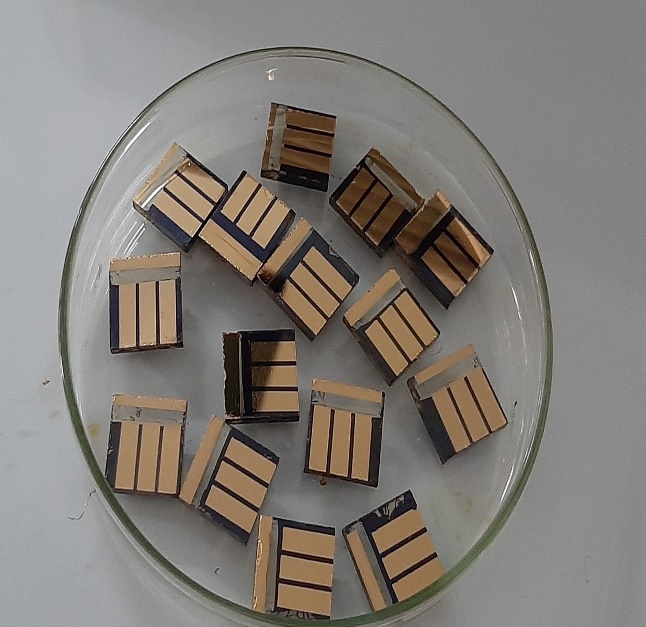


**Fig. S3**. The photograph images of the perovskite layers coated on CT-TiO_2_ (left) and perovskite solar cells based on CT-TiO_2_ ETLs (right).


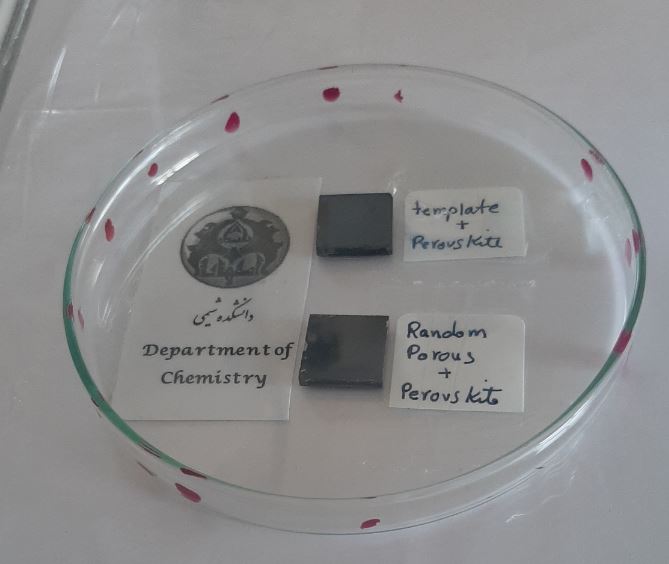

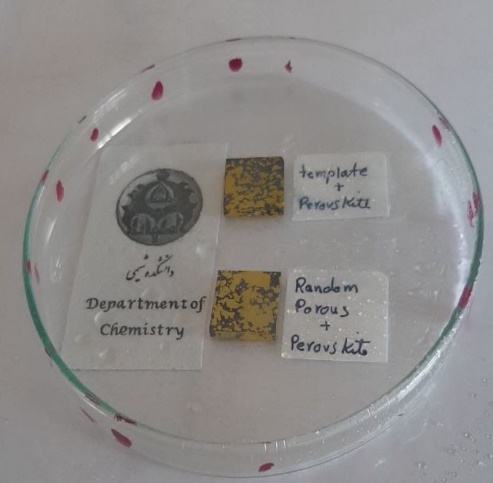


**First**

**When water was sprayed**


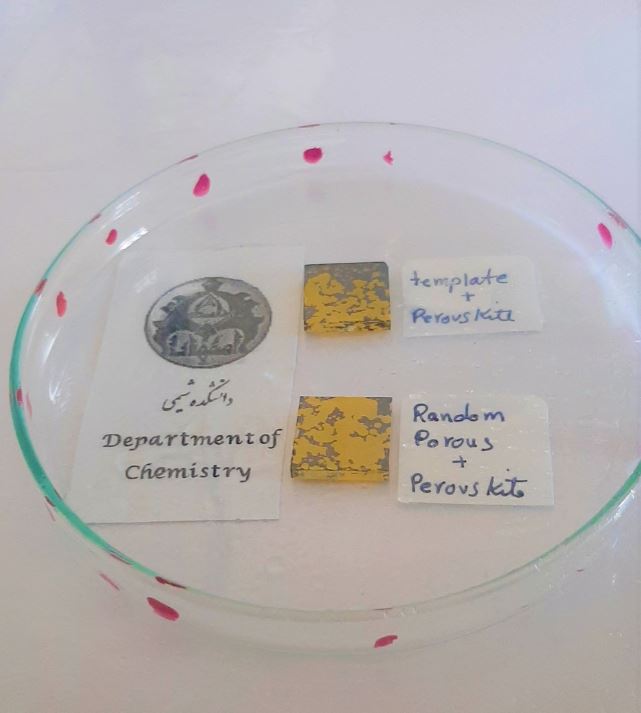

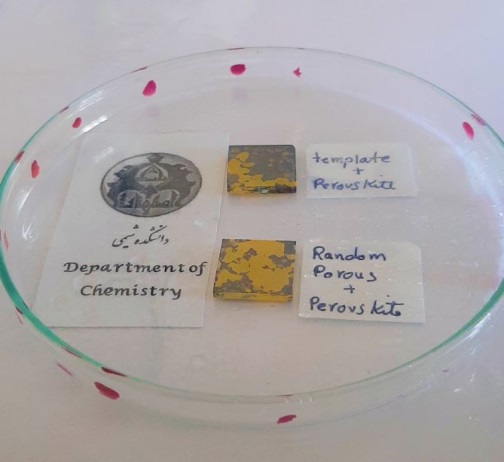

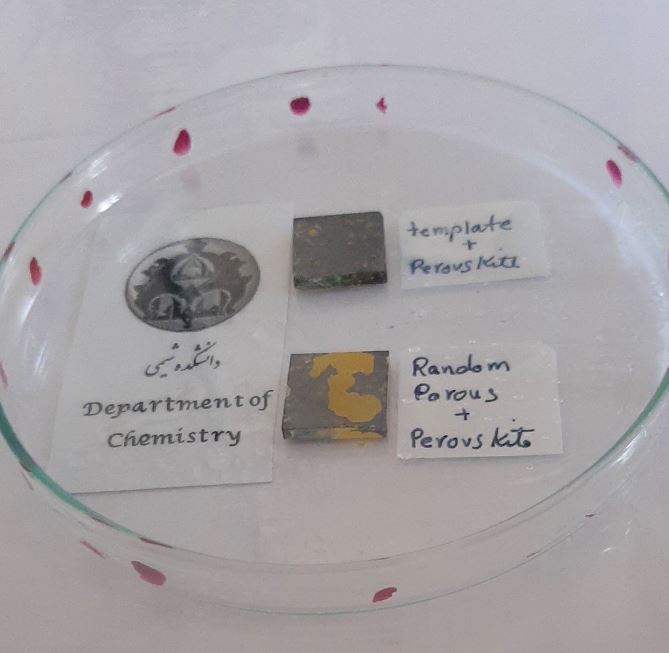


**After 6 min**

**After 3 min**

**After 1 min**

**Fig. S4**. Healing tests on FTO/CT-TiO_2_/Perovskite and FTO/RP-TiO_2_/Perovskite


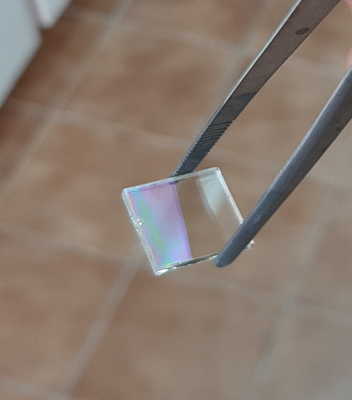

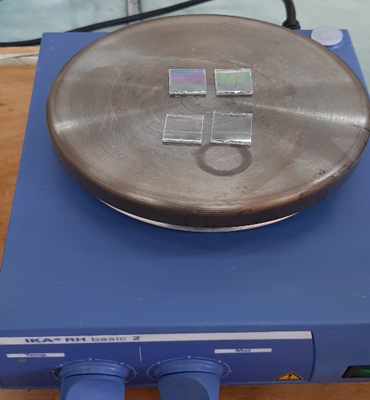


**1 & 2layer CT-TiO_2_**

**1layer NC-TiO_2_**

**Fig. S5**. FTO glass images with 1 layer CT-TiO_2_ (left), and (b) 1 and 2 layer CT-TiO_2_ and 1layer RP-TiO_2_ (right).

**Table S1.** Average PV data of three CIS-PSCs with CT and RP TiO_2_ ETLs

| Average | Jsc | Voc | FF | Efficiency% |
| --- | --- | --- | --- | --- |
| 1layer,speed:48 | 21.41$\pm$0.6 | 0.98$\pm$0.01 | 0.496$\pm$0.02 | 10.41$\pm$0.3 |
| 1layer,speed:30 | 17.17$\pm$0.6 | 0.925$\pm$0.004 | 0.492$\pm$0.019 | 7.81$\pm$0.2 |
| 1layer,speed:20 | 16.9$\pm$0.8 | 0.91$\pm$0.07 | 0.422$\pm$0.022 | 6.49$\pm$0.24 |
| 1layer,speed:10 | 12.71$\pm$0.62 | 0.7$\pm$0.02 | 0.459$\pm$0.02 | 7.68$\pm$0.2 |
| PR-TiO_2_  (standard) | 15.92$\pm$0.9 | 0.98$\pm$0.01 | 0.617$\pm$0.01 | 9.61$\pm$0.4 |


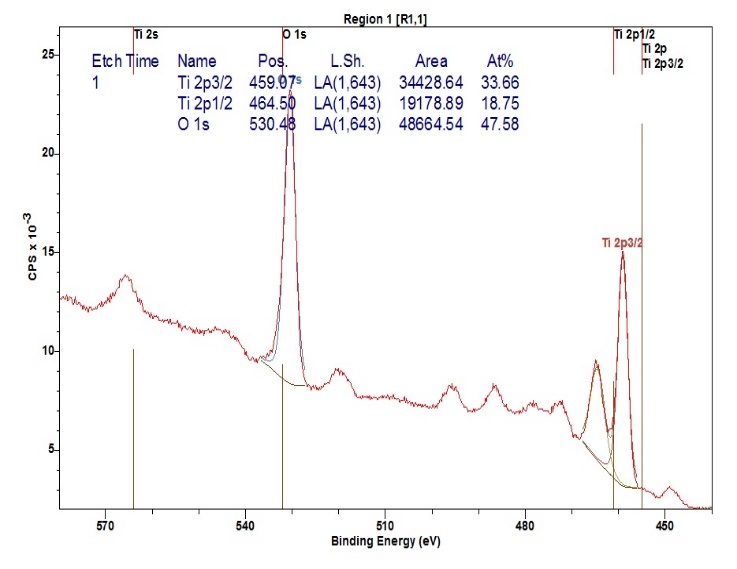

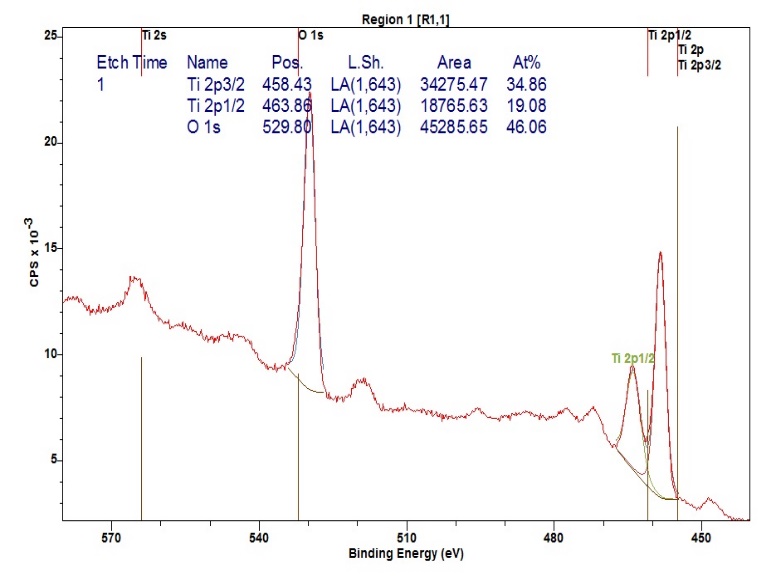


**Fig. S6**. XPS spectra of as prepared CT-TiO_2_ (left), and RP-TiO_2_ (right) with 46.06 O 1s At% for CT-TiO_2_ and 47.58% O 1s At% for RP-TiO_2_.


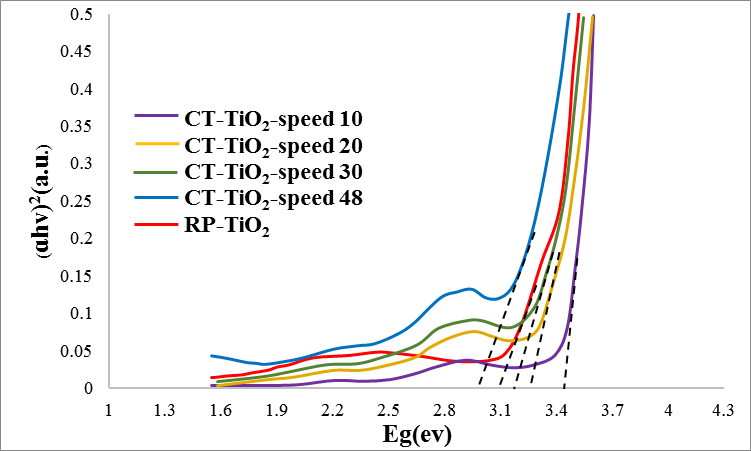


**Fig.S7**.Tauc plot for determining the band gaps. The band gap values of 2.90, 3.0, 3.15, 3.20, and 3.45 were obtained for CT-TiO_2_-speed 48, RP-TiO_2_, CT-TiO_2_-speed 30, CT-TiO_2_-speed 20, and CT-TiO_2_-speed 10, respectively


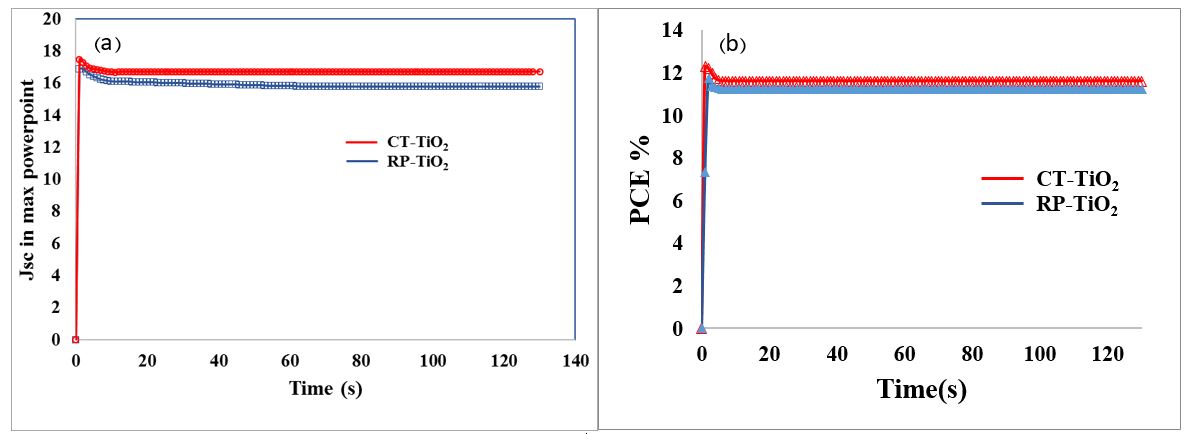


**Fig.S8**.(a)the photocurrent density and (b) stabilized efficiency of CIS-PSCs biased at maximum power points


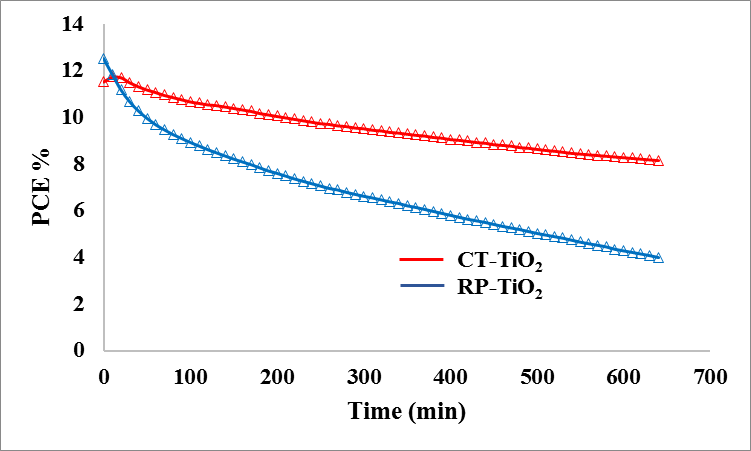


**Fig.S9**. The efficiency of CIS-PSC-based CT-TiO_2_ and RP-TiO_2_ under light-soaking

**Table S2**. Calculated FWHM of the CT and RP TiO_2_ layers

| ETL | FWHM (Peak 101) A⁰ |
| --- | --- |
| 3 layer CT | 0.519 |
| 2-layer CT | 0.419 |
| 1-layer CT | 0.276 |
| 1-layer RP | 0.161 |


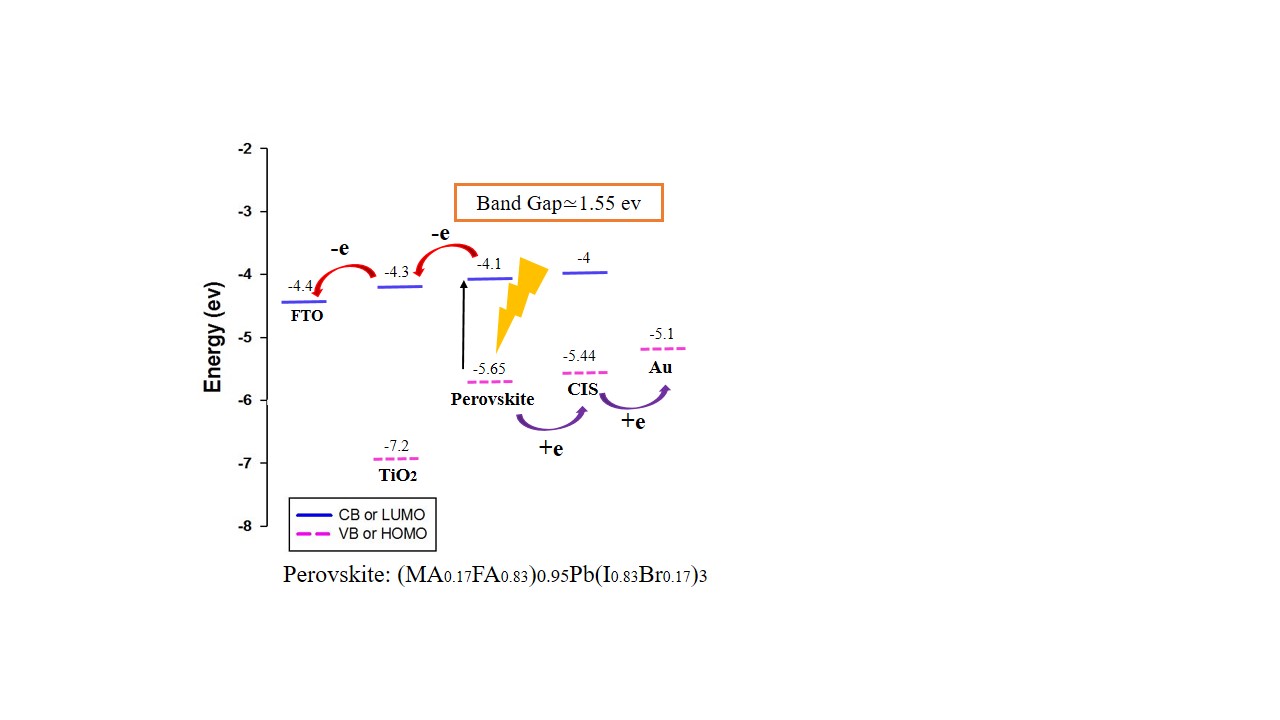


**Fig.S10**.The energy level diagram of the CIS-PSCs


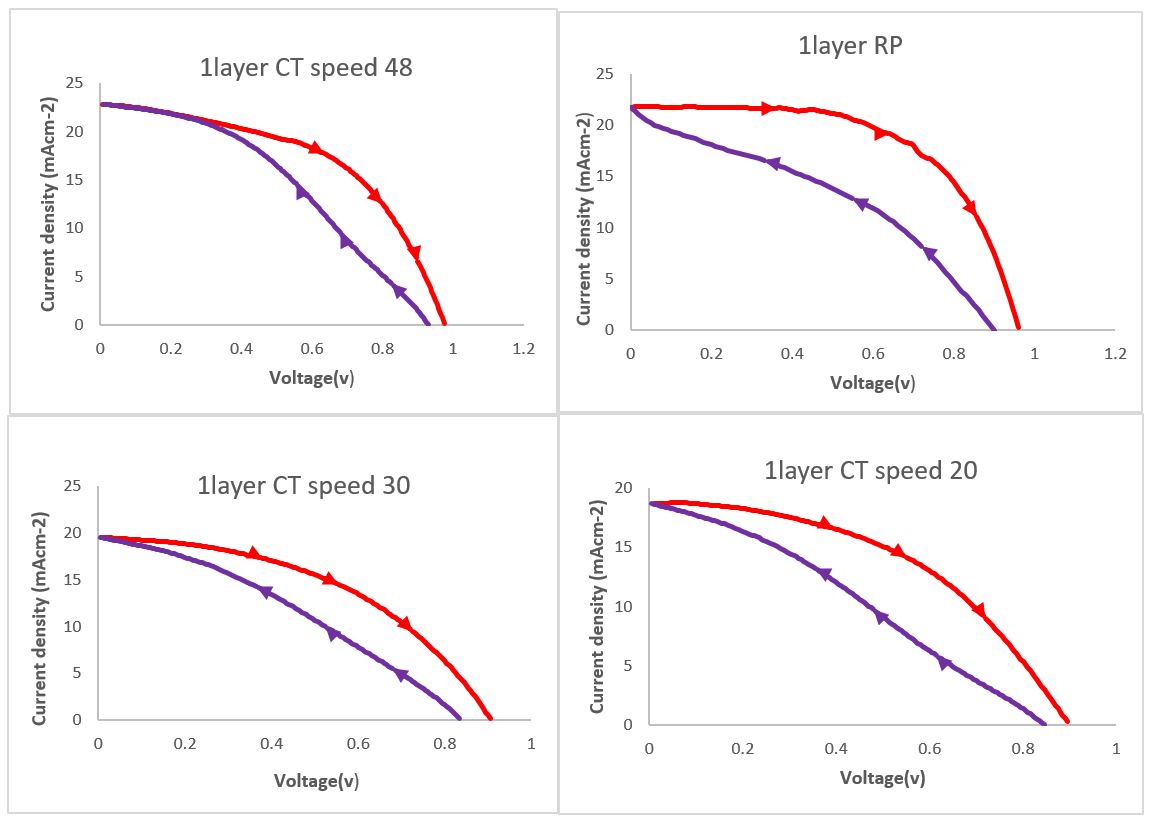


**Fig.S11**. Hysteresis effect investigation of the CIS-PSCs based on CT and RP TiO_2_

**Table S3**. Hysteresis index (HI) of CIS-PSCs based on CT and RP TiO_2_

| ETL | HI |
| --- | --- |
| 1-layer,speed:48 | 0.38 |
| 1-layer,speed:30 | 0.466 |
| 1-layer,speed:20 | 0.6 |
| Standard RP-TiO_2_ | 0.75 |
